# Identifying colorectal cancer caused by biallelic *MUTYH* pathogenic variants using tumor mutational signatures

**DOI:** 10.1038/s41467-022-30916-1

**Published:** 2022-06-06

**Authors:** Peter Georgeson, Tabitha A. Harrison, Bernard J. Pope, Syed H. Zaidi, Conghui Qu, Robert S. Steinfelder, Yi Lin, Jihoon E. Joo, Khalid Mahmood, Mark Clendenning, Romy Walker, Efrat L. Amitay, Sonja I. Berndt, Hermann Brenner, Peter T. Campbell, Yin Cao, Andrew T. Chan, Jenny Chang-Claude, Kimberly F. Doheny, David A. Drew, Jane C. Figueiredo, Amy J. French, Steven Gallinger, Marios Giannakis, Graham G. Giles, Andrea Gsur, Marc J. Gunter, Michael Hoffmeister, Li Hsu, Wen-Yi Huang, Paul Limburg, JoAnn E. Manson, Victor Moreno, Rami Nassir, Jonathan A. Nowak, Mireia Obón-Santacana, Shuji Ogino, Amanda I. Phipps, John D. Potter, Robert E. Schoen, Wei Sun, Amanda E. Toland, Quang M. Trinh, Tomotaka Ugai, Finlay A. Macrae, Christophe Rosty, Thomas J. Hudson, Mark A. Jenkins, Stephen N. Thibodeau, Ingrid M. Winship, Ulrike Peters, Daniel D. Buchanan

**Affiliations:** 1 Colorectal Oncogenomics Group, Department of Clinical Pathology, The University of Melbourne, Parkville, VIC 3010 Australia; 2 University of Melbourne Centre for Cancer Research, Victorian Comprehensive Cancer Centre, Parkville, VIC 3010 Australia; 3 Public Health Sciences Division, Fred Hutchinson Cancer Research Center, Seattle, WA USA; 4 Melbourne Bioinformatics, The University of Melbourne, Carlton, VIC Australia; 5 Ontario Institute for Cancer Research, Toronto, ON Canada; 6 Division of Clinical Epidemiology and Aging Research, German Cancer Research Center (DKFZ), Heidelberg, Germany; 7 Division of Cancer Epidemiology and Genetics, National Cancer Institute, National Institutes of Health, Bethesda, MD USA; 8 Division of Preventive Oncology, German Cancer Research Center (DKFZ) and National Center for Tumor Diseases (NCT), Heidelberg, Germany; 9 German Cancer Consortium (DKTK), German Cancer Research Center(DKFZ), Heidelberg, Germany; 10 Department of Epidemiology and Population Health, Albert Einstein College of Medicine, Bronx, NY USA; 11 Division of Public Health Sciences, Department of Surgery, Washington University School of Medicine, St Louis, MO USA; 12 Alvin J. Siteman Cancer Center at Barnes-Jewish Hospital and Washington University School of Medicine, St. Louis, MO USA; 13 Division of Gastroenterology, Department of Medicine, Washington University School of Medicine, St. Louis, MO USA; 14 Clinical and Translational Epidemiology Unit, Massachusetts General Hospital and Harvard Medical School, Boston, MA USA; 15 Division of Gastroenterology, Massachusetts General Hospital and Harvard Medical School, Boston, MA USA; 16 Channing Division of Network Medicine, Brigham and Women’s Hospital and Harvard Medical School, Boston, MA USA; 17 Broad Institute of MIT and Harvard, Cambridge, MA USA; 18 Department of Epidemiology, Harvard T.H. Chan School of Public Health, Boston, MA USA; 19 Department of Immunology and Infectious Diseases, Harvard T.H. Chan School of Public Health, Harvard University, Boston, MA USA; 20 Division of Cancer Epidemiology, German Cancer Research Center (DKFZ), Heidelberg, Germany; 21 University Medical Centre Hamburg-Eppendorf, University Cancer Centre Hamburg (UCCH), Hamburg, Germany; 22 Center for Inherited Disease Research (CIDR), Department of Genetic Medicine, Johns Hopkins University School of Medicine, Baltimore, MD USA; 23 Department of Medicine, Samuel Oschin Comprehensive Cancer Institute, Cedars-Sinai Medical Center, Los Angeles, CA USA; 24 Department of Preventive Medicine, Keck School of Medicine, University of Southern California, Los Angeles, CA USA; 25 Division of Laboratory Genetics, Department of Laboratory Medicine and Pathology, Mayo Clinic, Rochester, MN USA; 26 Lunenfeld Tanenbaum Research Institute, Mount Sinai Hospital, University of Toronto, Toronto, ON Canada; 27 Department of Medical Oncology, Dana-Farber Cancer Institute, Boston, MA USA; 28 Department of Medicine, Brigham and Women’s Hospital, Harvard Medical School, Boston, MA USA; 29 Cancer Epidemiology Division, Cancer Council Victoria, Melbourne, VIC Australia; 30 Centre for Epidemiology and Biostatistics, Melbourne School of Population and Global Health, The University of Melbourne, Melbourne, VIC Australia; 31 Precision Medicine, School of Clinical Sciences at Monash Health, Monash University, Clayton, VIC Australia; 32 Institute of Cancer Research, Department of Medicine I, Medical University Vienna, Vienna, Austria; 33 Nutrition and Metabolism Branch, International Agency for Research on Cancer, World Health Organization, Lyon, France; 34 Department of Biostatistics, University of Washington, Seattle, WA USA; 35 Division of Gastroenterology & Hepatology, Mayo Clinic, Rochester, MN USA; 36 Oncology Data Analytics Program, Catalan Institute of Oncology-IDIBELL, L’Hospitalet de Llobregat, Barcelona, Spain; 37 CIBER Epidemiología y Salud Pública (CIBERESP), Madrid, Spain; 38 Department of Clinical Sciences, Faculty of Medicine, University of Barcelona, Barcelona, Spain; 39 ONCOBEL Program, Bellvitge Biomedical Research Institute (IDIBELL), L’Hospitalet de Llobregat, Barcelona, Spain; 40 Department of Pathology, College of Medicine, Umm Al-Qura University, Mecca, Saudi Arabia; 41 Program in MPE Molecular Pathological Epidemiology, Department of Pathology, Brigham and Women’s Hospital, Harvard Medical School, Boston, MA USA; 42 Cancer Immunology Program, Dana-Farber Harvard Cancer Center, Boston, MA USA; 43 Department of Epidemiology, University of Washington, Seattle, WA USA; 44 Research Centre for Hauora and Health, Massey University, Wellington, New Zealand; 45 Department of Medicine and Epidemiology, University of Pittsburgh Medical Center, Pittsburgh, PA USA; 46 Departments of Cancer Biology and Genetics and Internal Medicine, Comprehensive Cancer Center, The Ohio State University, Columbus, OH USA; 47 Parkville Familial Cancer Centre, Royal Melbourne Hospital, Parkville, VIC Australia; 48 Colorectal Medicine and Genetics, Royal Melbourne Hospital, Parkville, VIC Australia; 49 Genomic Medicine and Family Cancer Clinic, Royal Melbourne Hospital, Parkville, VIC Australia; 50 Envoi Specialist Pathologists, Brisbane, QLD Australia; 51 University of Queensland, Brisbane, QLD Australia; 52 Department of Medicine, The University of Melbourne, Parkville, VIC Australia

**Keywords:** Colorectal cancer, Cancer genomics, Cancer genetics, Statistical methods

## Abstract

Carriers of germline biallelic pathogenic variants in the *MUTYH* gene have a high risk of colorectal cancer. We test 5649 colorectal cancers to evaluate the discriminatory potential of a tumor mutational signature specific to *MUTYH* for identifying biallelic carriers and classifying variants of uncertain clinical significance (VUS). Using a tumor and matched germline targeted multi-gene panel approach, our classifier identifies all biallelic *MUTYH* carriers and all known non-carriers in an independent test set of 3019 colorectal cancers (accuracy = 100% (95% confidence interval 99.87–100%)). All monoallelic *MUTYH* carriers are classified with the non-*MUTYH* carriers. The classifier provides evidence for a pathogenic classification for two VUS and a benign classification for five VUS. Somatic hotspot mutations *KRAS* p.G12C and *PIK3CA* p.Q546K are associated with colorectal cancers from biallelic *MUTYH* carriers compared with non-carriers (*p* = 2 × 10^−23^ and *p* = 6 × 10^−11^, respectively). Here, we demonstrate the potential application of mutational signatures to tumor sequencing workflows to improve the identification of biallelic *MUTYH* carriers.

## Abstract (web summary)

Germline biallelic pathogenic *MUTYH* variants predispose patients to colorectal cancer (CRC); however, approaches to identify *MUTYH* variant carriers are lacking. Here, the authors evaluated mutational signatures that could distinguish *MUTYH* carriers in large CRC cohorts, and found *MUTYH*-associated somatic mutations.

## Introduction

Genome-wide tumor profiling and associated computational approaches can provide a historical record of the mutational processes, both endogenous and exogenous, that were active during tumor initiation and progression, providing a tumor mutational signature (TMS) profile^1,2^. Several of these TMSs have been mechanistically shown to result from genetic defects related to homologous recombination repair deficiency^3^, DNA mismatch repair deficiency^4^, and base excision repair deficiency^5,6^, including in colorectal cancer (CRC)^7,8^. Therefore, TMSs can represent a functional manifestation of specific alterations in DNA repair pathways, with the potential application for not only identifying tumors caused by inherited defects in DNA repair genes but also providing functional evidence to support variant classification approaches in these DNA repair genes. The increasing application of tumor sequencing to identify targets for personalized therapy provides an opportunity to implement TMS analysis to gain additional clinically relevant knowledge on hereditary susceptibility earlier.

Identifying pathogenic variants in CRC and polyposis susceptibility genes has important implications for preventing subsequent primary cancers in the carrier^6,9,10^ and for the prevention of CRC in relatives through targeted screening approaches such as colonoscopy with polypectomy. The most common recessively inherited CRC and polyposis susceptibility genes include *MUTYH*^11,12^, and *NTHL1*^6,13^. Germline carriers of biallelic pathogenic variants in the *MUTYH* gene are almost certain to develop CRC, although monoallelic carriers of a *MUTYH* pathogenic variant may have only a small increased risk of CRC^14^. Current indications for germline testing for *MUTYH* include >20 colonic adenomas, although the phenotype has been described as variable where some biallelic *MUTYH* carriers develop CRC without the associated polyposis, suggesting biallelic *MUTYH* carriers may be missed with this current approach^15^. Unlike Lynch syndrome, where DNA mismatch repair immunohistochemistry is used on tumor samples for triaging patients to identify pathogenic variant carriers, no tumor-based biomarkers or testing approaches are currently used in diagnostic pathology to triage people for identifying germline biallelic *MUTYH* carriers.

A TMS profile characteristic of biallelic inactivation of *MUTYH* has been described in CRC^5,16^ and in other cancer types^17^. In previous work we evaluated all the existing specific single base substitution (SBS) and indel (ID) TMS using whole-exome sequencing of CRCs, demonstrating that the SBS TMSs, SBS18, and SBS36, when combined were the dominant TMSs in CRCs from biallelic *MUTYH* pathogenic variant carriers^7^. To support the application of SBS18 and SBS36 in the clinical setting, further evidence related to the accuracy of this approach is needed, particularly when applied to targeted panel sequencing data. Furthermore, our previous work generated the hypothesis that a combined SBS18 and SBS36 TMS could be applied to support the classification of germline *MUTYH* variants of uncertain clinical significance (VUS).

In this study, we: (1) evaluate the performance of SBS18 and SBS36 TMSs to identify germline biallelic pathogenic variant carriers and classify variants in the *MUTYH* gene in a large series of CRCs from the Genetic Epidemiology of Colorectal cancer Consortium (GECCO) tested with custom-designed targeted tumor sequencing assays, and (2) identify somatic mutation associations with biallelic *MUTYH* carriers within the somatic mutation landscape of CRCs.

## Results

### Distribution and classifications of CRCs across the study

The germline and somatic variants identified in *MUTYH* from all 5649 CRCs assessed in this study are summarized in Supplementary Fig. 1. Each tumor was categorized into one of five groups based on carriership of *MUTYH* variants and their classification as pathogenic, benign, or VUS (further defined in Supplementary Table 1): (1) *MUTYH* positives: tumors in people found to be germline carriers of two pathogenic variants (compound heterozygotes) or a homozygous pathogenic variant in *MUTYH*; (2) *MUTYH* monoallelics: tumors with only one germline heterozygous pathogenic variant in *MUTYH* and no other germline potential pathogenic variants; (3) *MUTYH* negatives: tumors with no germline or somatic pathogenic or potential pathogenic variants identified in *MUTYH*; (4) potential *MUTYH* biallelics: tumors in people homozygous for a germline potentially pathogenic variant or with two variants classified as either pathogenic or potentially pathogenic, but not two pathogenic variants; and (5) *MUTYH* uncertain*:* tumors in people with only one heterozygous potentially pathogenic or heterozygous somatic pathogenic variant.

**Fig. 1:**
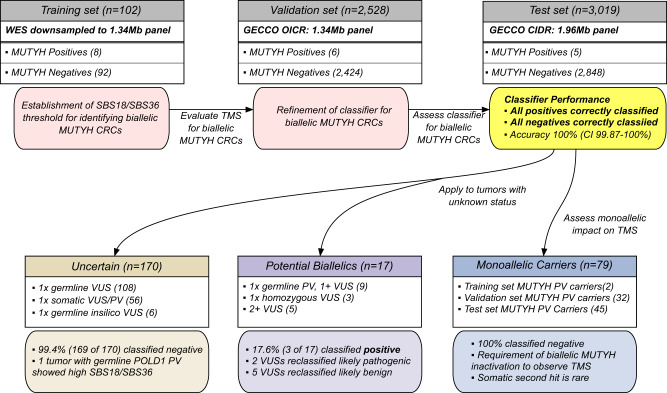
Overview of the analysis steps and groups of CRC tumor sequencing data included in the study, totaling 5649 CRCs. The SBS18/SBS36 TMS threshold was established using 102 CRCs down-sampled from whole-exome sequenced (WES) to intersect with the 1.34 Mb capture used to sequence the CRC tumors in the validation set. The 2528 CRCs sequenced with 1.34 Mb capture as part of the validation set were used to refine the SBS18/SBS36 classifier by including the somatic mutation count and TMS reconstruction error. The accuracy of the refined classifier was assessed using 3019 CRC tumors sequenced with a 1.96 Mb capture as part of the test set. The refined classifier was subsequently applied to 79 CRCs from monoallelic *MUTYH* pathogenic variant carriers, and CRCs defined as potential *MUTYH* biallelics and *MUTYH* uncertain status to determine its utility in variant classification. CI confidence interval, CIDR Center for Inherited Disease Research, CRC colorectal cancer, GECCO Genetic Epidemiology of Colorectal cancer Consortium, Mb megabase, OICR Ontario Institute of Cancer Research, PV pathogenic variant, SBS single bases substitution, TMS tumor mutational signature, VUS variant of uncertain clinical significance.

The tumors assessed were derived from 18 studies (Supplementary Table 2). The study design and distribution of tumors into training, validation, and test sets are summarized in Fig. 1. The demographic and clinic-pathological characteristics of the 5649 CRCs by training, validation, and test sets, by tumor *MUTYH* classification, and by recruiting study are shown in Supplementary Tables 2–5, respectively.

### Pathogenic variants in *MUTYH*

**Fig. 2:**
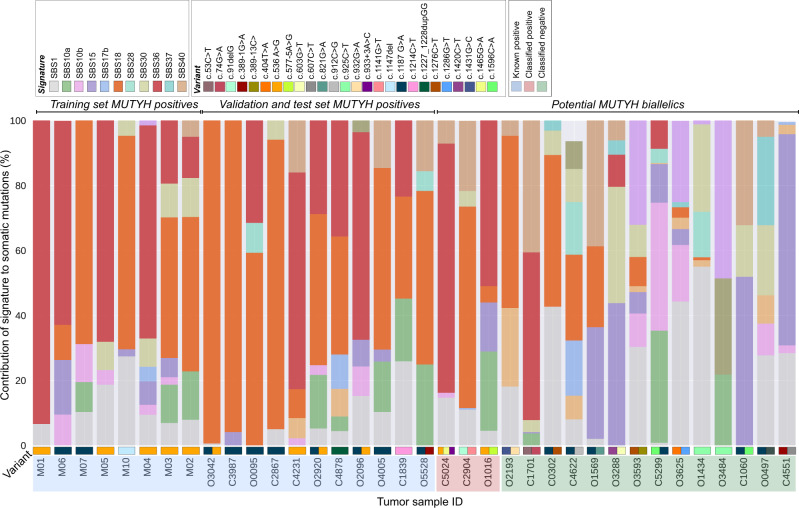
Observed tumor mutational signature profiles for 19 CRCs from germline biallelic *MUTYH* pathogenic variant carriers, and 17 CRCs carrying more than one *MUTYH* pathogenic or potentially pathogenic variant but not two pathogenic variants (potential biallelic). All the CRCs from the germline biallelic *MUTYH* PV carriers exhibit dominant SBS18 and/or SBS36 tumor mutational signature. Source data are provided as a Source Data file.

The pathogenic variants and clinicopathological characteristics of each of the 19 CRCs from biallelic *MUTYH* carriers are detailed in Supplementary Table 6. No *MUTYH* positive tumor showed microsatellite instability (MSI) according to MSIseq predictions. There were 79 monoallelic *MUTYH* pathogenic variant carriers and 17 potential *MUTYH* biallelics identified (Supplementary Table 7). Figure 2 summarizes the overall TMS profiles of the 19 *MUTYH* positive CRCs and the 17 CRCs from potential *MUTYH* biallelics (expanded to include all CRCs from *MUTYH* monoallelic carriers in Supplementary Fig. 2). Supplementary Fig. 3 and Supplementary Table 8 summarize the aggregated contexts and mutational signatures observed for each tumor class, respectively.

### SBS18/36 TMS threshold for identifying CRCs from *MUTYH* positives and its accuracy for discriminating *MUTYH* positives from *MUTYH* negatives

**Fig. 3:**
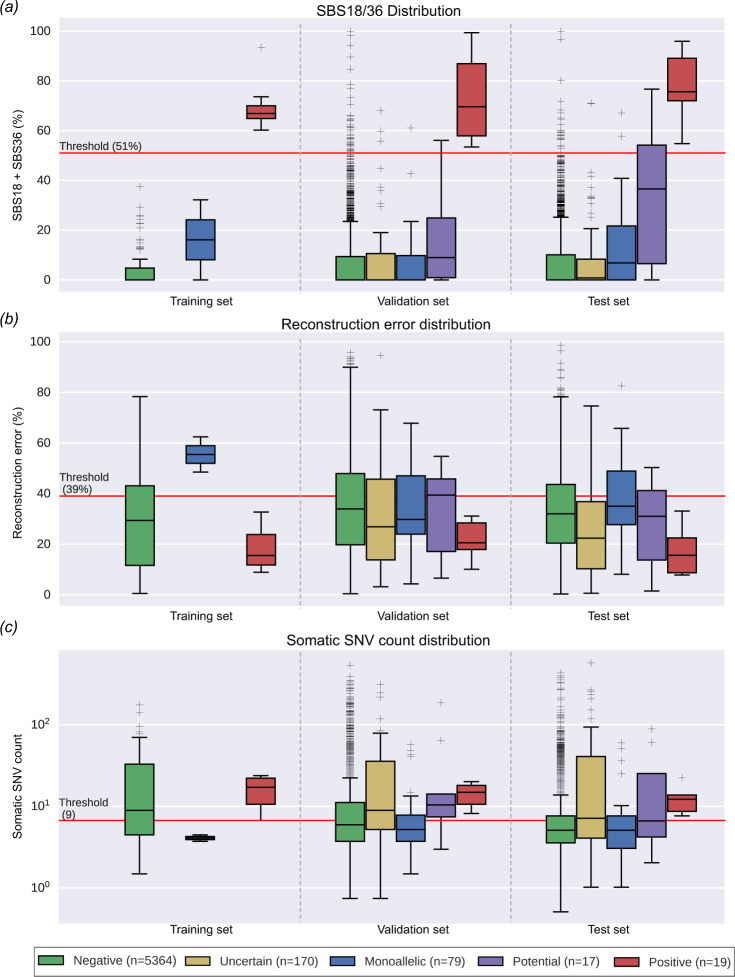
The distribution of SBS18/SBS36 tumor mutational signature, reconstruction error and somatic single nucleotide variant (SNV) count by the five tumor classification categories. Distribution of **a** SBS18/SBS36 tumor mutational signature, **b** tumor mutational signature reconstruction error, and **c** adjusted somatic SNV count across 5649 CRCs in the training set, validation set, and test set, grouped by germline pathogenic variant status (tumor classifications). The red horizontal line in each figure indicates the cut-offs that were determined based on the training set and validation set tumors. All boxes correspond to the 25th and 75th percentiles and the whiskers represent 1.5× the inter-quartile range (IQR) extending from the boxes. Lines at the middle of each box show the median. Individual observations are shown beyond the whiskers. Source data are provided as a Source Data file.

From the training set of 102 CRCs, including 8 *MUTYH* positive CRCs, we calculated the likelihood of biallelic *MUTYH* base excision repair deficiency TMS to be 95% when the sum of SBS18 and SBS36 exceeded 51% (range from 60.2 to 93.4%; Supplementary Table 9; Supplementary Fig. 4). We then assessed the accuracy of this baseline SBS18/36 classifier on the validation set of 2528 CRCs. All 6 *MUTYH* positives were correctly identified using the 51% SBS18/36 threshold, with no false negatives (Fig. 3a). Of the 2424 *MUTYH* negative CRCs, 45 were incorrectly classified as *MUTYH* positive and thus considered false positives. Therefore, the baseline classifier achieved 98.1% accuracy (95% confidence interval 97.5–98.6%), with 100% sensitivity (54.1–100%) and 98.1% specificity (97.5–98.6%) when applied to the validation set.

### The number of somatic mutations and degree of TMS reconstruction error are associated with false positive SBS18/36 TMS

**Fig. 4:**
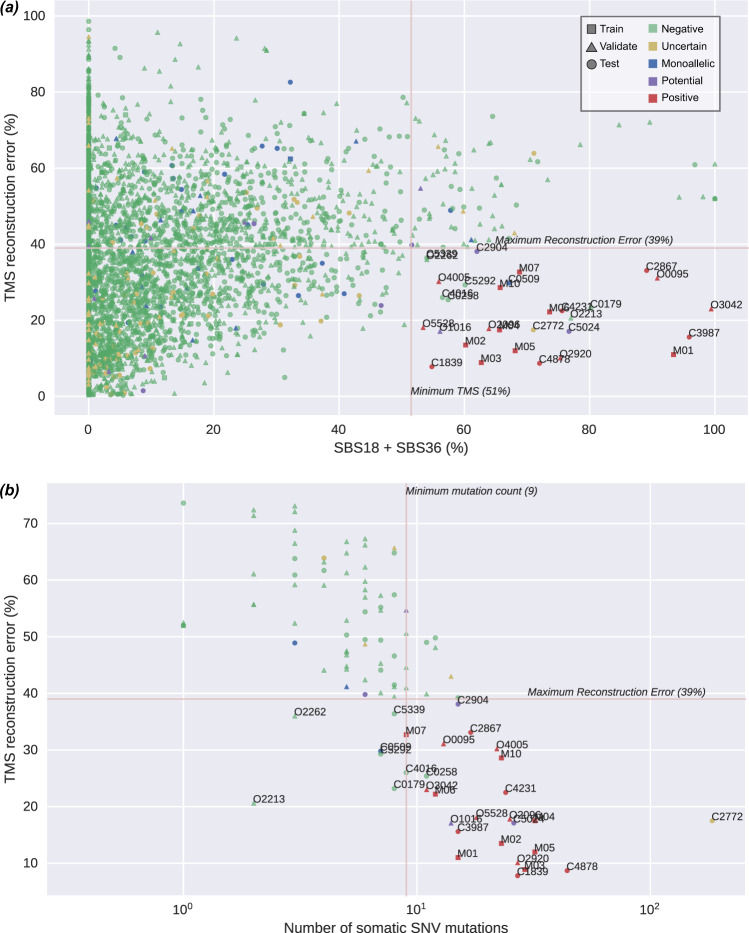
Distribution of SBS18/SBS36, somatic single nucleotide variants (SNVs), and tumor mutational signature (TMS) reconstruction error across CRCs from training, validation, and test sets. **a** The CRCs from the biallelic *MUTYH* pathogenic variant carriers cluster together based on high SBS18/SBS36 TMS and low TMS reconstruction error highlighting the need to include TMS reconstruction error in classifier, and **b** CRCs with greater than 95% likelihood of arising from biallelic *MUTYH* pathogenic variants based on TMS. The number of SNV mutations used in determining TMS (horizontal axis) and the TMS reconstruction error (vertical axis) demonstrates the importance of low reconstruction error (<39%) and sufficient somatic mutation count (≥9) for correctly classifying tumors from biallelic *MUTYH* pathogenic variant carriers (true positives). Source data are provided as a Source Data file.

We confirmed the absence of pathogenic variants in the 45 false positives by examining the sequencing data for any pathogenic variants that may have been overlooked by the variant calling pipeline. To determine features that could improve classification accuracy, we assessed each tumor’s somatic mutation count and TMS reconstruction error. The *MUTYH* positive CRCs from the training set (*n* = 8) and from the validation set (*n* = 6) exhibited a somatic mutation count ranging from 9 to 32 (mean ± SD 20.8 ± 7.8). In contrast, the 45 false positive CRCs from the validation set exhibited significantly lower somatic mutation counts, ranging from 1 to 12 (mean ± SD of 5.1 ± 2.6; *p* = 8 × 10^−17^, t-test). The 14 *MUTYH* positives from the training and validation sets exhibited reconstruction error ranging from 8.9 to 32.7% (mean ± SD 19.8 ± 8.3%), whereas the 45 false positive CRCs showed significantly higher reconstruction error ranging from 20.6 to 73.1% (mean ± SD 54.0 ± 11.5%; *p* = 1 × 10^−14^, t-test). By considering somatic mutation count and reconstruction error, the 45 false positives could be differentiated from the 14 *MUTYH* positives, evidenced by 43 of 45 CRCs (96%) having a reconstruction error >39%, and 40 of 45 (89%) having <9 somatic mutations (Figs. 3a–c, 4a, b). Combining these two constraints eliminated all false positives while still detecting all 14 *MUTYH* positives, providing an optimized *MUTYH* TMS classifier.

### Evaluating the optimized *MUTYH* TMS classifier on an independent test set of CRCs

We applied this optimized classifier, comprising SBS18 + SBS36 > 51%, reconstruction error <39%, and somatic mutation count ≥9, to the independent test set (*n* = 3019), with the somatic mutation counts adjusted for the differing panel sizes. All five *MUTYH* positives and all 2848 *MUTYH* negatives were correctly identified. This corresponds to 100% accuracy (95% CI 99.87–100%), sensitivity (47.8–100%), and specificity (99.87–100%) (Fig. 1), demonstrating the classifier’s likely generalizability to independent data.

### Classifying CRCs from *MUTYH* monoallelics and potential *MUTYH* biallelics

The SBS18/36 TMS was significantly higher in biallelic *MUTYH* carrier CRCs compared with both non-*MUTYH* carrier CRCs (*p* = 3 × 10^−112^, t-test) and monoallelic *MUTYH* pathogenic variant carrier CRCs (*p* = 5 × 10^−29^, t-test). When applying our optimized classifier, none of the 79 *MUTYH* monoallelics were classified as positive (Fig. 4a, b), demonstrating that monoallelic inactivation of *MUTYH* is insufficient to observe the SBS18/36 TMS in CRCs. To investigate somatic inactivation of the wildtype allele in the *MUTYH* monoallelics, we assessed loss of heterozygosity (LOH) as a potential second somatic event. Evidence of LOH across *MUTYH* was observed in 4% (224/5649) of CRCs in this study, but these tumors did not show significantly elevated SBS18/36. The 224 tumors with LOH spanning *MUTYH* were supported by 8.2 ± 7.2 mutations (mean ± sd) across the entire LOH region, with 1.8 ± 0.9 mutations within 100,000 bases of *MUTYH*. Public data suggests LOH does not commonly affect *MUTYH*: 0/60 Pan-Cancer Analysis of Whole Genomes (PCAWG) CRCs and 69/583 (12%) of The Cancer Genome Atlas (TCGA) CRCs showed evidence of copy number loss across *MUTYH*. Structural variants are similarly rare^18^. Four of the 79 (5%) *MUTYH* monoallelics exhibited LOH but none were classified as positive based on the classifier. Additionally, 61 tumors harbored pathogenic or potentially pathogenic somatic mutations in *MUTYH* across the entire cohort (1.1%), but no pathogenic somatic mutation in *MUTYH* was observed in any of the monoallelic CRCs, suggesting a second somatic event is a rare event in *MUTYH* monoallelic carriers. We did not observe any statistically significant association between SBS18/36 and tumor stage in the monoallelic or biallelic carriers (Supplementary Table 10 and Supplementary Fig. 5).

Given this differential in biallelic and monoallelic *MUTYH* carriers, we applied the optimized classifier to 17 potential *MUTYH* biallelic CRCs carrying more than one variant (germline or somatic) classified as either pathogenic or VUS to determine if the SBS18/36 TMS could provide functional evidence for biallelic inactivation and, therefore, support variant classification (Table 1). For two VUSs, p.G381W and c.577-5A>G, the TMSs provide support for pathogenicity (Table 1). Neither variant has been seen in gnomAD and have inconclusive computational predictions by REVEL and CADD, but the high observed TMS, in conjunction with acceptable reconstruction error, somatic mutation count, and no evidence for LOH, adds support for pathogenicity. Similarly, the high TMS observed in tumor C5024 suggests that one of these VUSs c.933+3A>C or p.A489T is likely to be pathogenic. For five VUSs, p.R426C, p.S304R, p.R274Q, p.R309C, and p.T477T, our classifier adds evidence suggesting that these variants are likely benign. In particular, p.R309C was homozygous in two independent tumors that the classifier predicted to be *MUTYH* negative. Participant O1569 carried the germline monoallelic pathogenic variant c.1187G>A p.G396D and a second germline variant c.821G>A p.R274Q classified as a VUS by ClinVar (REVEL 0.826; CADD 33). Previous studies suggest that c.R274Q mutant *MUTYH* has partial activity compared to wild-type protein^19,20^. In this tumor, ten somatic mutations were detected with high reconstruction error (45.8%) and SBS18/36 TMS of 24.9%—which suggests <1% likelihood of the tumor being related to biallelic *MUTYH* inactivation (Supplementary Table 9). This adds evidence that c.821G>A p.R274Q is likely benign.

**Table 1:** Participants categorized into the potential *MUTYH* biallelic group, based on either carrying a germline pathogenic variant and one or more VUSs, or multiple VUSs.

ID	AgeDx	Sex	Source	*MUTYH* variants	ClinVar	GnomAD	CADD	REVEL	SBS18/36 (%)	Error (%)	Somatic mutations	TMS-based prediction	Variant reclassification
C2904	70–79	F	Germline Germline	c.91delG p.A31PfsTer27 c.1141G>T p.G381W	PV VUS	None None	23.0 23.8	None 0.521	62	38.1	15	Positive	VUS->PV
O1016	40–49	F	Germline Germline	c.536A>G p.Y179C c.577-5A>G	PV VUS	0.001 None	24.7 16.9	0.963 None	56.1	17.1	14	Positive	VUS->PV
C5024	50–59	M	Germline Germline Germline	c.536A>G p.Y179C c.933+3A>C c.1465G>A p.A489T	PV VUS VUS	1.5 × 10^−3^ 6.5 × 10^−5^ 6.5 × 10^−5^	24.7 6.4 29.4	0.963 None 0.724	76.7	17.1	26	Positive	None
C0302	60–69	M	Germline Germline	c.1187G>A p.G396D c.1276C>T p.R426C	PV VUS	0.003 0.001	29.4 22.9	0.551 0.615	46.7	23.9	11	Negative	VUS->Benign
C4622	40–49	M	Germline Germline	c.1187G>A p.G396D c.912C>G p.S304R	PV VUS	0.003 3 × 10^−5^	29.4 12.8	0.551 0.229	26.4	45.4	9	Negative	VUS->Benign
O1569	30–39	F	Germline Germline	c.1187G>A p.G396D c.821G>A p.R274Q	PV VUS	0.003 0.0002	29.4 33	0.551 0.229	24.9	45.8	10	Negative	VUS->Benign
C5299	40–49	M	Germline Somatic	c.1187G>A p.G396D c.1596C>A p.F532L	PV VUS	0.003 2.8 × 10^−5^	29.4 12.3	0.551 0.063	8.7	1.5	175	Negative	None
C4551	40–49	F	Germline Somatic	c.389-1G>A c.926G>A p.R309H	PV VUS	1.2 × 10^−5^ 0.0005	2.3 13.9	0.592 0.293	0	3.6	120	Negative	None
O0497	70–79	F	Germline Somatic	c.1187G>A p.G396D c.607C>T p.R203C	PV VUS	0.003 3.7 × 10^−5^	29.4 23.9	0.551 0.358	0	4.6	19	Negative	None
C1060	70–79	M	Germline Germline	c.1465G>A p.A489T c.933+3A>C	VUS VUS	6.5 × 10^−5^ 6.5 × 10^−5^	6.4 29.4	None 0.724	0	50.3	4	Negative	None
O2193	60–69	F	Germline Germline	c.1431G>C p.T477T c.932G>A p.R311K	VUS VUS	0.006 2.6 × 10^−4^	4.6 5.3	0.039 0.24	53	54.7	9	Negative	None
O3288	80–89	F	Germline Germline	c.1420C>T p.R474C c.603G>T p.M201I	VUS VUS	3.2 × 10^−5^ None	23.2 16.8	0.546 0.262	9.9	41.7	10	Negative	None
O3593	60–69	F	Germline Germline	c.1276C>T p.R426C c.389-13C>G	VUS VUS	0.001 6.5E−5	22.9 14.9	0.615 None	9	10.7	251	Negative	None
O1434	60–69	M	Germline	c.925C>T p.R309C (H)	VUS	5.4 × 10^−4^	13.90	0.592	0.9	25.8	14	Negative	VUS->Benign
O3484	30–39	M	Germline	c.925C>T p.R309C (H)	VUS	5.5 × 10^−4^	13.90	0.592	0	39.4	4	Negative	VUS->Benign
C1701	30–39	F	Germline Germline Germline Germline	c.1431G>C p.T477T (H) c.74G>A p.G25D c.53C>T p.P18L c.165+37_1650+39delGTT	VUS VUS VUS VUS	0.006 1.1 × 10^−3^ 1.1 × 10^−3^ None	4.6 14.2 16.7 12.8	0.039 0.111 0.2 None	51.6	39.8	6	Negative	VUS->Benign
O3625	70–79	M	Somatic Somatic	c.1286G>T p.G429V c.404T>A p.V135D	None None	None None	22.3 28.9	0.658 0.898	3.2	6.6	86	Negative	None

The characteristics of participants and each of the variants identified including ClinVar classification, CADD and REVEL prediction scores, and gnomAD allele frequency, as well as the features of the optimized classifier: SBS18 + SBS36 (>51% for positivity), TMS reconstruction error (<39% for positivity), and somatic mutation count (≥9 for positivity) and the TMS-based pathogenicity prediction (positive for biallelic inactivation, negative for no biallelic inactivation). We indicate the seven VUSs that the classifier provides evidence for reclassification as either likely pathogenic or likely benign. *AgeDx* age of diagnosis, *PV* pathogenic variant, *TMS* tumor mutational signature, *VUS* variant of uncertain significance, (*H)* homozygous for germline variant; CADD score >20.0 or REVEL score >0.6 considered predicted pathogenic.

Of the 170 tumors in *MUTYH* uncertain group (Supplementary Table 7), 169 were classified as *MUTYH* negative by the classifier. The single positive tumor exhibited high mutational burden (93.7 mutations/megabase (Mb)) and was found to harbor a germline potentially pathogenic variant in *POLD1* (c.1225C>T p.R409W).

### Somatic mutation landscape of CRCs from biallelic *MUTYH* pathogenic variant carriers

To evaluate the impact of biallelic inactivation of *MUTYH* on the somatic mutational landscape, we combined all 19 *MUTYH* positive tumors across the three datasets. We previously observed that SBS18 and SBS36 are associated with specific pathogenic variants in *MUTYH*^7^. Specifically, homozygous pathogenic variants at the 5′ end of the gene (exons 1–10) tend to give rise to SBS36, while SBS18 is more prevalent in homozygous pathogenic variants at the 3′ end of the gene. Comparing homozygous p.Y179C tumors to p.G396D homozygous tumors, SBS18 and SBS36 were both significantly different between these two groups of tumors (*p* = 0.015 and 0.024, respectively, t-test; Supplementary Fig. 6). Three additional carriers with homozygotes near p.G396D (c.1214C>T p.P405L, c.1227_1228dupGG p.E410Gfs*43 and c.1147del p.A385PfsTer23) support the possibility of domain-specific TMSs. When aggregated with the p.G396D tumors, we see similarly significant differences between the TMSs (*p* = 0.011 and 0.012 respectively, t-test; Supplementary Table 6).

Under the definition that hypermutated tumors have >10 mutations/Mb^21^, 12/19 (63.1%) *MUTYH* positives were considered hypermutated (mean ± SD 22.0 ± 8.8 somatic mutations). None showed evidence of MSI or somatic *POLE* exonuclease domain mutations. In comparison, 469 (10.4%) of the 4510 microsatellite stable *MUTYH* negative tumors were considered either hypermutated (*n* = 415) or ultra-hypermutated (*n* = 54) (>100 mutations/Mb^21^), representing a significant difference (*p* = 4 × 10^−8^, binomial test) (Fig. 3c).

**Fig. 5:**
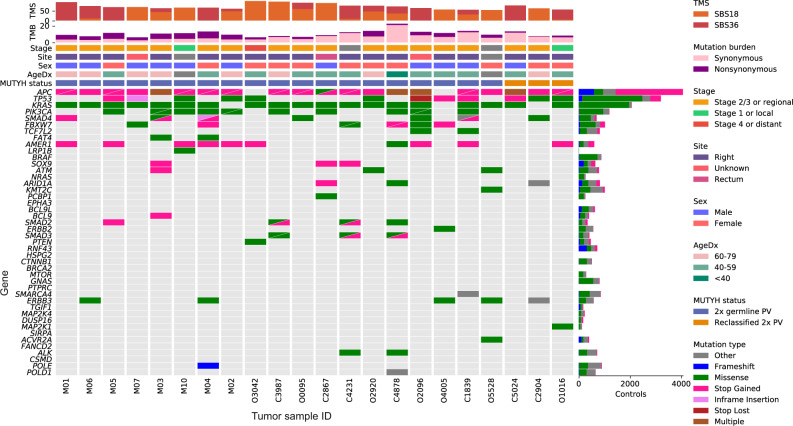
Somatic mutation landscape of the 19 CRCs from biallelic *MUTYH* pathogenic variant carriers, as well as the 3 CRCs from carriers of variants of uncertain clinical significance that were reclassified as likely pathogenic in this study. The 40 most commonly mutated CRC genes^48^ are included, as well as known CRC genes *ALK*, *CSMD1*, *POLE*, and *POLD1*. *KRAS* was found to be significantly more commonly mutated in our biallelic *MUTYH* carrier CRCs. Source data are provided as a Source Data file. AgeDx age of diagnosis, TMB tumor mutational burden (mutations/Mb), TMS tumor mutational signature.

Somatic mutations were compared between the 19 *MUTYH* positives and 5,352 *MUTYH* negatives (Fig. 5; expanded to include *MUTYH* monoallelic tumors in Supplementary Fig. 7). Several genes were found to have a significant enrichment of non-synonymous mutations in the *MUTYH* positives, including *KRAS*, *PIK3CA*, and *AMER1* (Table 2), consistent with previous findings^16^. In *KRAS* and *PIK3CA*, a substantial proportion of all mutations could be attributed to specific individual mutations: p.G12C (*KRAS*) and p.Q546K (*PIK3CA*). We demonstrated the utility of these hotspot mutations on smaller panels, showing that they identify most biallelic carriers, though with lower sensitivity and specificity than can be achieved using a larger panel that incorporates SBS18/36 TMS (Supplementary Table 11). Both mutations were found to be mutation types highly specific to the SBS18 and SBS36 mutational trinucleotide contexts, supporting a link to the DNA damage profile associated with biallelic *MUTYH* inactivation. Similarly, the proportion of somatic mutations attributable to SBS18/36, measured as relative likelihood^22^, was higher in all enriched genes (Table 2), adding evidence that the association between *MUTYH* positives and these genes has a mechanistic basis.

**Table 2:** Significantly enriched individual somatic mutations, as well as genes significantly affected by non-synonymous somatic mutations, observed in more than two *MUTYH* positive tumors.

Gene	Variant (context)	*MUTYH* positives	*MUTYH* negatives	*p*-value	SBS18/36 relative likelihood (*MUTYH* positives vs *MUTYH* negatives)
*KRAS*	c.34G>T p.G12C (CCA>A)	16/19 (84%)	127/5364 (2.4%)	2 × 10^−23^	62%
*PIK3CA*	c.1636C>A p.Q546K (GCA>A)	7/19 (37%)	36/5364 (0.7%)	6 × 10^−11^	83%
*KRAS*	Gene-wide	17/19 (89%)	2025/5364 (38%)	5 × 10^−6^	58% vs 17%
*AMER1*	Gene-wide	9/19 (47%)	592/5364 (11%)	8 × 10^−5^	35% vs 12%
*PIK3CA*	Gene-wide	10/19 (53%)	934/5364 (17%)	5 × 10^−4^	60% vs 12%
*ROBO2*	Gene-wide	3/19 (16%)	55/5364 (1.0%)	1 × 10^−3^	42% vs 20%
*TAF1L*	Gene-wide	5/19 (26%)	420/5364 (8%)	0.01	36% vs 13%
*SMAD4*	Gene-wide	6/19 (32%)	638/5364 (12%)	0.02	36% vs 13%
*SMAD2*	Gene-wide	4/19 (21%)	308/5364 (6%)	0.02	53% vs 13%
*APC*	Gene-wide	17/19 (89%)	3468/5364 (65%)	0.03	45% vs 18%
*ERBB3*	Gene-wide	4/19 (21%)	388/5352 (7%)	0.045	47% vs 13%

Somatic mutations observed in the significantly enriched genes in MUTYH positives were more often associated with the trinucleotide contexts related to the SBS18/36 tumor mutational signatures (TMS) as measured by the SBS18/36 relative likelihood. *P*-values were calculated with Fisher’s exact test (two-sided).

## Discussion

We previously demonstrated that combining *MUTYH*-related base excision repair deficiency mutational signatures SBS18 and SBS36 was more effective than each signature alone for identifying germline biallelic *MUTYH* carriers using whole exome sequencing of CRC tumors^7^. In this study, we trained, validated, and then tested the effectiveness of our *MUTYH* SBS18/36 TMS classifier for identifying CRCs from biallelic *MUTYH* pathogenic variant carriers in a large cohort of 5649 tumors that underwent targeted multi-gene panel sequencing from formalin-fixed paraffin-embedded (FFPE) tissue DNA. The addition of somatic mutation count and TMS reconstruction error to the SBS18/36 threshold enabled the determination and validation of classifier parameters, namely SBS18/36 TMS proportion >51%, TMS reconstruction error <39%, and somatic mutation count ≥9, that yielded 100% accuracy for distinguishing *MUTYH* positives from *MUTYH* negatives when applied to an independent dataset. Furthermore, when the *MUTYH* TMS classifier was applied to a group of potential *MUTYH* biallelics as a functional approach to evaluate the pathogenicity of VUSs, we found support for two VUSs, p.G381W and c.577-5A>G, being likely pathogenic, while for five VUSs, p.S304R, p.R274Q, p.R426C, p.R309C, and p.T477T, our classifier provided evidence they were likely benign. Finally, we provided a detailed view of the somatic mutation landscape of CRCs from biallelic *MUTYH* pathogenic variant carriers based on a consensus set of 205 cancer genes, identifying specific mutations in *KRAS* and *PIK3CA* genes that were associated with CRC tumorigenesis in biallelic *MUTYH* carriers.

### Effectiveness of TMSs to identify biallelic *MUTYH* carriers from targeted panel sequencing data

We demonstrated that the SBS18/36 TMS was robust when scaling down from a whole exome capture (67 Mb)^7^ to a 1.34 Mb capture in the training set. Furthermore, SBS18/36 remained highly correlated between the different capture sizes of the validation (1.34 Mb, *ρ* = 0.904) and test (1.96 Mb, *ρ* = 0.911) sets when compared with the whole exome capture (Supplementary Table 12). This is important for the generalizability and implementation of this *MUTYH* TMS classifier approach where tumor sequencing for clinical diagnostics is still largely embedded with targeted multi-gene panel testing rather than whole exome or whole genome sequencing. Developing and applying the classifier parameters on different capture sizes and assays (validation set = 1.34 Mb and test set = 1.96 Mb) while still achieving 100% accuracy supports the potential for a broad application of this approach to different clinical panels in use globally.

### Resolving false positives in the TMS data

Despite demonstrating that the combined SBS18/36 TMS was effective at identifying CRCs from biallelic *MUTYH* carriers, the reduction in capture size from exome to the 1.34 Mb targeted panel required the inclusion of justifiable constraints in our classifier to eliminate false positives. By considering the number of observed somatic variants and the TMS reconstruction error, all 24 false positives observed in the independent dataset of 3022 CRCs were eliminated. Although the number of somatic mutations is a critical factor influencing the accuracy of reported TMSs, the literature lacks consensus recommending minimum mutation counts, with estimates ranging from 200^23^, 100^24^, 50^25^, down to 5^26^. We showed that the presence of either of the two hotspot mutations *KRAS* p.G12C or *PIK3CA* p.Q546K resulted in 89.5% sensitivity (area under the curve 0.932) for detecting *MUTYH* positive CRCs, representing the lower limit of detection. For the 1.34 Mb capture, we found that tumors with reconstruction error >39% or carrying <9 somatic mutations were unlikely to generate a SBS18/36 TMS profile that was caused by biallelic inactivation of *MUTYH*. These measures are negatively correlated (*ρ* = −0.41) and exclude tumors for different reasons: the constraint on minimum somatic mutations reflects our previous finding that *MUTYH* positive CRCs exhibit significantly higher tumor mutational burden (TMB) than *MUTYH* negative mismatch repair (MMR)-proficient tumors^7^, confirmed by this larger study. The constraint on reconstruction error eliminates tumors with TMSs that are not strongly supported by the observed mutations. Increasing capture size tends to increase both mutation count and reduce TMS reconstruction error (Supplementary Tables 12 and 13) which will aid in reducing false positives and the resolution of cases that fall close to the current classifier thresholds. Calibration of the *MUTYH* TMS classifier for custom captures that are unique to individual diagnostic laboratories may be required for effective implementation.

### Application to variant classification

We identified several key findings that support the incorporation of our *MUTYH* TMS classifier in variant classification approaches, mirroring the multifactorial approach adopted when classifying MMR variants:^27,28^ (1) Biallelic inactivation of *MUTYH* is necessary for generation of the SBS18/36 TMS, providing functional evidence of defective base excision repair, (2) the presence of the SBS18/36 TMS is a very strong predictor with 100% accuracy, (3) low false positive rate when TMS reconstruction error and somatic mutation count is added to the classifier for targeted panel sequencing data, and (4) somatic inactivation of *MUTYH* rarely occurs as evidenced by the rarity of second somatic hits in *MUTYH* monoallelics and no biallelic somatic inactivation was observed in 5649 CRCs.

Based on these key observations, the *MUTYH* TMS classifier supported pathogenicity for two VUSs and an absence of support for pathogenicity for five VUS. The *MUTYH* TMS classifier supported pathogenicity for at least one of c.933+3A>C and c.1465G>A p.A489T variants, although further work is needed to determine which one is or if they occur on a haplotype. Although the presence of the SBS18/36 TMS provides strong evidence for pathogenicity, the absence of the SBS18/36 TMS in supporting a likely benign classification should be considered with other factors, namely, the possibility the VUS is on the same allele as the pathogenic variant (in *cis*) and that we currently do not know if there is variability in deleterious effects of different pathogenic variants within *MUTYH* that result in a less dominant SBS18/36 TMS phenotype. Our findings support the application of the *MUTYH* TMS classifier as a tool to aid in variant classification approaches for *MUTYH*, and may help resolve some of the 58% (689 of 1190) of variants in *MUTYH* in ClinVar that are classified as either uncertain or with a conflicting classification.

### Somatic landscape and segregation of SBS18 and SBS36

Evidence is accumulating that the two signatures, SBS18 and SBS36, segregate based on the *MUTYH* domain affected by the variant^7^: the presence of the c.1187G>A p.G396D pathogenic variant contributes predominantly to the SBS18 signature, while c.536A>G p.Y179C contributes predominantly to SBS36. Although SBS18 and SBS36 are similar signatures (cosine similarity 0.91) characterized by C>A transversions, they differ substantially in specific contexts: GCA>A, CCA>A, and ACA>A. This suggests that the affected domain alters tumor etiology, which could help us better understand the biology of tumors that arise and potentially inform clinical decision making. For example, both significantly enriched somatic mutations in *KRAS* c.34G>T p.G12C (CCA>A) and *PIK3CA* c.1636C>A p.Q546K (GCA>A) found in the *MUTYH* positives (Table 2) are in variant contexts that differ significantly between signatures, suggesting domain-specific hotspots that may inform treatment decision making.

The finding of commonly occurring specific somatic mutations and mutated genes has treatment implications. Cross-referencing the significant biomarkers found in this study with existing clinical actionability databases^29^ identified relevant drug associations, including FDA guidelines suggesting likely resistance to Cetuximab and Panitumumab (*KRAS* p.G12C), and pre-clinical trials suggesting responsiveness to MEK, ERK, BCL-XL, IGF-1R, PI3K pathway inhibitors, and BH3 mimetics. Further, clinical trials with direct inhibitors of the *KRAS* p.G12C allele^30^ are ongoing in CRC and represent a promising potential therapy for *MUTYH* positives. The FDA approval of the PD-1 inhibitor, pembrolizumab, as a therapy for tumors with TMB greater than 10^31^ is also clinically relevant, with our results indicating that most *MUTYH* positives are hypermutated (despite being MMR-proficient/microsatellite stable).

### Limitations

We cannot exclude the possibility that other mechanisms may cause SBS18/36 TMS that are more difficult to detect using panel sequenced data, such as LOH or structural variants. We could not determine the impact tumor heterogeneity might have on TMS. This might be more impactful for *MUTYH* monoallelic carriers, where somatic inactivation of the wildtype allele may occur later in tumorigenesis, however, overall we found no significant increase in the SBS18/36 TMS for *MUTYH* monoallelic carrier CRCs compared with *MUTYH* negative CRCs (10.8 ± 15.4% v. 7.1 ± 12.4%, *p* = 0.45, t-test) supporting previous findings that monoallelic *MUTYH* pathogenic variants alone do not result in loss of base excision repair^7^. Doublet and indel signatures were not considered for this study due to low numbers in panel-sequenced data. The majority of our *MUTYH* positives carry the most common *MUTYH* pathogenic variants—by expanding the analysis to different ethnic groups and a broader diversity of *MUTYH* variants we can improve the generalizability of the *MUTYH* TMS classifier and potentially classify a greater number of *MUTYH* variants. Similarly, the application to non-CRCs needs to be investigated with the aim of developing a tumor agnostic *MUTYH* TMS classifier.

In conclusion, identifying germline biallelic *MUTYH* carriers is important for personalized surveillance and cancer prevention in carriers and cancer risk prediction in relatives. The variable clinical phenotype, lack of tumor-based screening to triage CRC-affected patients for *MUTYH* gene testing (akin to MMR immunohistochemistry for Lynch syndrome), conflicting reports regarding CRC risks in monoallelic *MUTYH* carriers, and the absence of validated functional assays for variant classification present important clinical challenges that limit effective identification and clinical management of *MUTYH* carriers. Key findings from this study address these current limitations, namely, the high accuracy of the tumor-based *MUTYH* TMS classifier for identifying biallelic *MUTYH* pathogenic variants and the absence of SBS18/36 TMS in *MUTYH* monoallelics enabled its application to variant classification; we re-classified seven germline VUSs, including supporting a likely pathogenic classification for two variants, c.1141G>T p.G381W and c.577-5A>G. The significantly enriched somatic mutations in *KRAS* c.34G>T p.G12C and *PIK3CA* c.1636C>A p.Q546K in *MUTYH* positive CRCs, where both mutations correspond to dominant contexts in SBS18/36, support a direct connection to *MUTYH*-related base excision repair deficiency and provide potential biomarkers for targeted therapy. With the increasing use of tumor sequencing for precision oncology and clinical diagnostics, our findings support the incorporation of our *MUTYH* TMS classifier into clinical tumor sequencing workflows as an accurate method to identify biallelic *MUTYH* pathogenic variant carriers, particularly when biallelic *MUTYH* status is not suspected, or when germline testing fails to yield a high-confidence resolution due to VUSs or conflicting results. Finally, the incorporation of analyses directed towards TMS for identifying hereditary subtypes could improve the detection of carriers and efforts to provide precision prevention of CRC.

## Methods

### Study participants

All participants provided written informed consent, and each study was approved by the relevant research ethics committee or institutional review board. The University of Melbourne Human Research Ethics Committee approved this research (study IDs 1750748, 1954921). Three independent sets of CRC-affected individuals (Fig. 1) were included in the study: (1) a training set of 102 CRCs with whole-exome sequencing from the Australasian Colon Cancer Family Registry (ACCFR; *n* = 47)^32,33^ and the ANGELS study (*n* = 55)^7^; (2) a validation set of 2906 CRCs from GECCO sequenced at the Ontario Institute for Cancer Research with a 1.34 Mb targeted panel covering 205 genes;^34^ and (3) a test set of 3093 CRCs and advanced adenomas from GECCO and sequenced at the Center for Inherited Disease Research with a 1.96 Mb targeted panel covering 350 genes. DNA was extracted from FFPE CRCs and matched with germline tissue (either blood-derived or normal mucosa). A description of each of the studies and the breakdown of the CRCs are provided in Supplementary Tables 2–5.

### Tumor sequencing analysis

The mean coverage of *MUTYH* across the capture regions for the training, validation, and test tumor datasets was 581.2 ± 156.9, 753.9 ± 578.0, and 1542.5 ± 1176.8, respectively (mean ± SD) (Supplementary Fig. 8). For the training data, somatic variant calls were generated from the intersection of Strelka v2.9.2^35^ and Mutect2^36^, with minimum tumor sequencing depth of 25 reads and variant allele fraction of 10%. Variant calls were then limited to the same 1.34 Mb capture region as the validation set. For the panel-sequenced validation and test sets, somatic variants were generated from the intersection of Strelka v1.0.1547 and Mutect, as per^34^ (see Supplementary Methods for more detail). Tumors with at least one somatic single nucleotide variant (SNV) were included for analysis, which comprised 102, 2528, and 3019 tumors in the training, test, and validation sets, respectively, for a total of 5649 tumors assessed in this study (Fig. 1).

LOH in the tumor across *MUTYH* was determined by identifying germline heterozygous variants with homozygous somatic equivalents (see Supplementary Methods)^4^. Copy number loss was assessed in PCAWG and TCGA CRC cohorts with available consensus data^37^ and copy number segment data^38^, respectively (see Supplementary Methods). TMB was calculated as the combined number of SNVs, insertions, and deletions (indels) per megabase of capture sequence. MSI status was determined using the method described by MSIseq^39^. Reported transcript and protein changes in *MUTYH* refer to NM_001128425.1 and NP_001121897.1 respectively.

### Germline *MUTYH* variant calling

The mean coverage of *MUTYH* across the capture regions for the training, test, and validation germline datasets was 372.0 ± 118.1, 280.4 ± 352.6, and 425.7 ± 321.5 respectively (mean ± SD) (Supplementary Fig. 8). Germline variants in the test and validation datasets were called using Strelka^35^ and limited to PASS calls with a minimum depth of 50 reads and a minimum variant allele fraction of 10%.

### Variant Classifications

Variants classified by ClinVar^40^ as likely pathogenic or pathogenic were grouped and considered “pathogenic” for the purposes of this study (*n* = 18 unique variants). Variants of uncertain significance or with conflicting interpretations in ClinVar and/or variants that were predicted by computational metrics as pathogenic were retained and defined as “potentially pathogenic” variants (*n* = 105 unique variants) (Supplementary Fig. 1). The variant classification methods are detailed in the Supplementary Methods. The classified variants were then used to classify all tumors into five categories (Fig. 1 and Supplementary Table 1).

### Tumor mutational signature (TMS) generation

TMSs were calculated for each of the 5649 CRCs using the simulated annealing method described by SignatureEstimation^41^, an approach previously applied successfully to panel-sequenced data^42^. The pre-defined set of Catalog of Somatic Mutations in Cancer (COSMIC) mutational signatures v3.1^43^ was reduced to a set of 14 signatures previously observed in 59 whole-genome sequenced CRCs as determined in PCAWG^1^, including the known base excision repair signatures SBS18 and SBS36 associated with defective *MUTYH*^16^ and SBS30 associated with defective *NTHL1*^6,44^. The TMS reconstruction error measures how accurately a reported signature profile reflects the observed mutations and was calculated as the cosine distance between the observed mutational context counts and the predicted mutational context counts computed from the mutational signatures^45^. We used the Python (v3.7.4) SciPy (v1.4.1)^46^ implementation of simulated annealing (“basinhopping”) to calculate the linear combination of TMSs that minimized reconstruction error.

### Determining SBS18/36 TMS thresholds for identifying *MUTYH* positive CRCs

From the training set, 8 CRCs from known *MUTYH positives* and 92 confirmed *MUTYH* negatives were used to establish a combined SBS18 and SBS36 TMS threshold for identifying CRCs from biallelic *MUTYH* carriers that were specific to the targeted 1.34 Mb/205 gene panel (as previously applied to whole exome sequencing data^7^).

### Predicting biallelic *MUTYH* carriers from the validation and test sets of CRCs and evaluating the accuracy of TMSs

Based on the combined SBS18/36 TMS threshold calculated from the training set of 100 CRCs, we predicted the *MUTYH* status of the validation set of CRCs and assessed its accuracy against the tumor classifications based on variant calling. The TMS-based classifier was then optimized using the validation set, by considering the number of somatic mutations and the TMS reconstruction error in addition to the SBS18/36 TMS threshold. The test set was then utilized as an independent dataset to assess the accuracy of the optimized classifier. The test set somatic mutation count was compared to the classifier threshold after adjusting by the proportional difference in panel sizes (1.34/1.96). To further assess the classifier’s utility for *MUTYH* variant classification, we applied it to CRCs defined as *MUTYH* monoallelics, potential *MUTYH* biallelics, and *MUTYH* uncertain (Fig. 1).

### Statistical analyses

All statistical analyses were performed using Python 3.7.4. NumPy 1.17.3^47^ was used for numerical calculations. Statistical calculations were performed using SciPy 1.4.1^46^. All t-tests were performed as two-sided and assuming equal variance with all p-values reported unadjusted unless otherwise specified.

### Reporting summary

Further information on research design is available in the Nature Research Reporting Summary linked to this article.

## Supplementary information

Supplementary Information

Reporting Summary

## Data Availability

All data generated in this study are included in this published article (and its supplementary information files/Source Data file). The original panel-sequenced data used in this study are available at the database of Genotypes and Phenotypes (dbGaP). The Ontario Institute of Cancer Research (OICR) data is available under accession code phs002050.v1.p1. The Center for Inherited Disease Research (CIDR) data is available under accession code phs001905.v1.p1. The whole exome sequencing data used in this study has been previously published^7^. This data is available from the Colon Cancer Family Registry via a “request to collaborate with the CCFR” application process (www.coloncfr.org/collaboration). Colorectal Adenocarcinoma TCGA copy number data was downloaded from cBioPortal (https://www.cbioportal.org/) using the data sequenced in the Colorectal Adenocarcinoma (TCGA, PanCancer Atlas) study. Copy number loss was assessed in the PCAWG with the consensus copy number data downloaded from https://dcc.icgc.org/releases/PCAWG/consensus_cnv. Mutational signature definitions were downloaded from the COSMIC website at https://cancer.sanger.ac.uk/signatures/downloads/. Source data are provided with this paper.

## Source data

Source Data
