# Supplementary material for: Identifying colorectal cancer caused by biallelic MUTYH pathogenic variants using tumor mutational signatures
Source: Nat Commun. 2022 Jun 6;13:3254. doi: 10.1038/s41467-022-30916-1 (PMC9170691; doi:10.1038/s41467-022-30916-1)
Supplement: Supplementary file 1 — Supplementary Information [file 41467_2022_30916_MOESM1_ESM.pdf]

SUPPLEMENTARY METHODS

Study Participants

The study population included men and women diagnosed with incident invasive primary colon or rectal cancer (CRC) that were enrolled in one of the following participating studies (Supplementary Table 2):

Colorectal Cancer Family Registry (CCFR)[1]

The CCFR is an NCI-supported consortium consisting of six centers dedicated to the establishment of a comprehensive collaborative infrastructure for interdisciplinary studies in the genetic epidemiology of colorectal cancer. The CCFR includes data from approximately 42,500 total subjects in 15,000 families (10,500 probands, and 26,770 unaffected and affected relatives and 4,276 unrelated controls and 923 spouse controls). Cases and controls, ages 20 to 74 years, were recruited at the six participating centers beginning in 1998. Between 1999 and 2002, female cases and controls 50-74 years enrolled into the Seattle CCFR (SCCFR) were subsequently enrolled in a complementary study of post-menopausal hormone use and CRC risk (PMH). CCFR and PMH implemented a standardized questionnaire that is administered to all participants, and includes established and suspected risk factors for colorectal cancer, which includes questions on medical history and medication use, reproductive history (for female participants), family history, physical activity, demographics, alcohol and tobacco use, and dietary factors. This study selected tumor samples from population-based cases in the following population-based centers: Seattle (including a subset of PMH), Ontario (OFCCR), and Australia (ACCFR).

ANGELS (Applying Novel Genomic approaches to Early-onset and suspected Lynch Syndrome colorectal and endometrial cancers)

The ANGELS study was established in 2017 through NHMRC project grant funding (GNT1125269). Participants diagnosed from 2015 onwards were recruited to the study from Family Cancer and Genetics Clinics across Australia and New Zealand if they met one of four recruitment criteria; 1) individual diagnosed with a DNA mismatch repair (MMR)-deficient colorectal or endometrial cancer categorized as suspected Lynch syndrome, with an absence of a germline MMR gene pathogenic variant and no evidence of tumor *MLH1* promoter methylation; 2) Individual diagnosed with an early-onset colorectal cancer (EOCRC; ≤45 years) that was MMR-proficient; 3) individual diagnosed with MMR-proficient colorectal cancer and a family history that fulfils Amsterdam I criteria (otherwise known as Familial Colorectal Cancer Type X or FCCTX); and 4) individual carrying a germline variant classified as a variant of uncertain clinical significance in either the MMR (*MLH1*, *MSH2*, *MSH6* or *PMS2*), *POLE*, *POLD1*, *MUTYH* or *NTHL1* genes. At the time of enrollment, medical records and pedigree information were provided by clinics and participants completed a mailed self-administered questionnaire including information on demographic, medical, diet, and lifestyle factors. Peripheral blood sample and formalin-fixed paraffin embedded tissue were collected from each participant. For this study, only participants meeting criteria 2 (EOCRC) were included in the analysis.

Colorectal Cancer Study of Austria (CORSA)[2]

In the ongoing CORSA study, more than 16,000 Caucasian participants have been recruited within the province-wide screening project "Burgenland Prevention Trial of Colorectal Disease with Immunological Testing" (B-PREDICT) since 2003. All inhabitants of the Austrian province Burgenland aged between 40 and 80 years are annually invited to participate in fecal immunochemical testing and haemoccult positive screening participants are invited for colonoscopy. CORSA participants have been recruited in the four KRAGES hospitals in Burgenland, Austria, and additionally, at the Medical University of Vienna

(Department of Surgery), the Viennese hospitals “Rudolfstiftung” and the “Sozialmedizinisches Zentrum Süd”, and at the Medical University of Graz (Department of Internal Medicine).

American Cancer Society Cancer Prevention Study II (CPSII)[3,4]

The CPS-II Nutrition cohort is a prospective study of cancer incidence and mortality in the United States, established in 1992. At enrollment, participants completed a mailed self-administered questionnaire including information on demographic, medical, diet, and lifestyle factors. Follow-up questionnaires to update exposure information and to ascertain newly diagnosed cancers were sent biennially starting in 1997. Reported cancers were verified through medical records, state cancer registry linkage, or death certificates. This study was conducted with Institutional Review Board approval.

Cancer Risk Assessment Study (CRA)[5]

All individuals who had surgery at the Mayo Clinic Rochester MN, Methodist Hospital Rochester MN, or St. Mary’s Hospital Rochester MN, who consented to participate from 1995 to 1998 were included in this study’s collection. Every patient was offered a chance to participate, no exclusion criteria were applied. Subjects who consented were given a form to complete that contained questions about lifestyle, medical history, and family history. Materials collected from subjects included peripheral blood, resected tumor from the center and the rim of the neoplasm, as well as normal colon both adjacent to the tumor and at the surgical margin. One representative piece from each was flash frozen and stored at -70C. The remaining piece was fixed in formalin and embedded in paraffin. Additionally, if lymph nodes or metastatic tumors were present, material was collected for these as well.

Colorectal Cancer Genetics & Genomics (CRCGEN, CRCGEN_2)

This Spanish study combines data from three case-control studies. The first one, performed in University Hospital of Bellvitge, L'Hospitalet, Barcelona, recruited incident pathology-confirmed CRC cases during the period 1996-1998. The second study was performed in the same hospital during the period 2007-2015 and the third study was conducted in Hospital of Leon, Leon, during 2008-2013. This study included both colorectal cancer cases and adenoma cases. Adenoma or serrated polyps were detected at screening colonoscopy. Patients with high-risk lesions were selected, defined as ≥ 5 adenomas/serrated polyps, or ≥ 1 adenoma/serrated polyp ≥ 20mm.

Darmkrebs: Chancen der Verhütung durch Screening (DACHS)[6,7]

This German study was initiated as a large population-based case-control study in 2003 in the Rhine-Neckar-Odenwald region (southwest region of Germany) to assess the potential of endoscopic screening for reduction of colorectal cancer (CRC) risk and to investigate etiologic determinants of disease, particularly lifestyle/environmental factors and genetic factors. Cases with a first diagnosis of invasive CRC (International Classification of Diseases 10 codes C18-C20) who were at least 30 years of age (no upper age limit), German speaking, a resident in the study region, and mentally and physically able to participate in a one-hour interview, were recruited by their treating physicians either in the hospital a few days after surgery, or by mail after discharge from the hospital. Cases were confirmed based on histologic reports and hospital discharge letters following diagnosis of CRC. All hospitals treating CRC patients in the study region participated. Based on estimates from population-based cancer registries, more than 50% of all potentially eligible patients with incident CRC in the study region were included. During an in-person interview, data were collected on demographics, medical history, family history of CRC, and various life-style factors, as were blood and mouthwash samples. Formalin-fixed, paraffin-embedded, surgical specimens of

CRC patients were collected from cooperating pathology institutes and transferred to the tissue bank of the National Center for Tumor Diseases in Heidelberg.

Hispanic Colorectal Cancer Study (HCCS)

HCCS is a population-based study of individuals self-identified as Hispanic with a diagnosis of colorectal cancer. Cases are identified from the California Cancer Registry or directly from local hospitals in the Los Angeles region [LAC + USC County Hospital and University of Southern California (USC) Norris Comprehensive Cancer Center]. All men and women over 21 years of age with a first-time diagnosis of CRC (ICD-O-3 codes: C18–C21) after January 1, 2008, were eligible for participation. Risk factor and dietary questionnaires, pathology reports, and saliva samples (for genotyping) were collected using methodologies developed in the Colon Cancer Family Registry and the Multiethnic Cohort (MEC). Participants recruited into the HCCS were born in Mexico, the US, Central/South America, Cuba, the Caribbean Islands, or Europe. The present study includes # cases with formalin-fixed paraffin-embedded (FFPE) colorectal tumor tissue available.

Health Professionals Follow-up study (HPFS)[8,9]

The HPFS cohort comprises over 51,000 men aged 40-75 years at enrollment and followed since the study started in 1986. Participants provided information on health-related exposures, including current and past smoking history, weight, height, diet, supplement use, alcohol intake, physical activity, aspirin use, endoscopy procedures, and family history of colorectal cancer every two years (or four years for diet) through questionnaires. Colorectal cancer and other outcomes were reported by participants or next-of-kin and were followed up through review of the medical and pathology record by physicians. Lethal unreported colorectal cancer cases were identified (and confirmed) through next-of-kin, use of the National Death Index and medical record review. Overall, more than 97% of self-reported

colorectal cancers were confirmed by medical record review. Information was abstracted on histology, primary tumor location, TNM staging, tumor size and multiplicity, and the number of positive and negative lymph nodes. In 1993–1995, over 18,000 participants mailed blood samples by overnight courier, which were aliquoted into buffy coat and stored in liquid nitrogen. In 2001–2004, nearly 14,000 participants who had not provided a blood sample previously mailed in a swish-and-spit sample of buccal cells. FFPE tissue blocks were collected from hospitals where participants with colorectal carcinoma had undergone tumor resection or endoscopic biopsy (for pre-operatively treated rectal cancer). The study pathologist (S.O.) reviewed hematoxylin-and-eosin (H&E)-stained tissue sections and recorded histopathological features. The study protocol was approved by the institutional review boards of the Brigham and Women's Hospital and Harvard T.H. Chan School of Public Health, and those of participating registries as required.

Iowa Women's Health Study (IWHS)[10,11]

In the IWHS, a 16-page baseline questionnaire was completed and returned by 41,836 randomly selected women, ages 55 to 69 years, who resided in Iowa and held a valid driver's license at baseline in 1986. Comprehensive self-reported demographic, dietary, lifestyle, and medication data were collected during the baseline IWHS evaluation (1986). Incident colorectal cancer cases were identified through annual linkage with the Iowa Cancer Registry, which is a member of the National Cancer Institute's Surveillance, Epidemiology, and End Results (SEER) program. Colorectal cancer cases were identified using International Classification for Diseases in Oncology (ICD-O) codes. Beginning in 2006, archived, paraffin-embedded tissue specimens were requested from incident colorectal cancer cases diagnosed through December 31, 2002.

Melbourne Collaborative Cohort Study (MCCS)[12]

The MCCS is a prospective cohort study of 41,513 healthy adult volunteers between the ages of 27 and 76 years (99% aged 40-69) recruited from the Melbourne metropolitan area between 1990 and 1994. By 31 December 2009, 1,046 participants had a first histopathological diagnosis of invasive adenocarcinoma of the colon or rectum identified by a record linkage to the Victorian Cancer Registry following the baseline study visit. Beginning in 2004, archived formalin-fixed paraffin embedded archived tissue specimens were requested from incident colorectal cancer cases diagnosed through 1995 to 2009. All CRC cases eligible for this study were selected based on the availability of a tumor sample and having no pre-baseline history of CRC (as confirmed by the Victorian Cancer Registry).

Nurses' Health Study (NHS)[13]

The NHS cohort began in 1976 when over 121,000 female registered nurses ages 30 to 55 years returned the initial questionnaire that ascertained a variety of important health-related exposures. Colorectal cancer and other outcomes were reported by participants or next-of-kin and followed up through review of the medical and pathology record by physicians. Overall, more than 97% of self-reported colorectal cancers were confirmed by medical-record review. Participants have been sent questionnaires biennially to update information on lifestyle factors and newly diagnosed disease. Data on histology and primary location were abstracted. FFPE tissue blocks were collected from hospitals where participants with colorectal carcinoma had undergone tumor resection or endoscopic biopsy (for pre-operatively treated rectal cancer).

Nurses' Health study II (NHSII)[14]

The Nurses' Health Study II is an ongoing cohort of over 116,000 female registered nurses in the US, aged 25-42 years at baseline in 1989. Demographic, lifestyle and health-related information were obtained from participants at baseline and updated every 2 years using self-

administered questionnaires. Study participants who had not previously reported a diagnosis of cancer and had responded to the 1995 study questionnaire were invited to provide blood samples between 1996 and 1999. Blood samples were collected from over 29,000 participants, aged 32 to 54 years at the time of blood draw. Similarly, between 2004 and 2006, active study participants who had not previously provided a blood sample were invited to provide buccal samples. Swish-and-spit samples of buccal cells were received from nearly 30,000 participants. Participants with a prior history of any cancer (except non-melanoma skin cancer), ulcerative colitis, or familial polyposis syndromes were excluded. FFPE tissue blocks were collected from hospitals where participants with colorectal carcinoma had undergone tumor resection or endoscopic biopsy (for preoperatively treated rectal cancer).

Prostate, Lung, Colorectal, and Ovarian Cancer Screening Trial (PLCO)[15]

PLCO is a large, randomized, two-arm trial that enrolled over 154,000 men and women between the age of 55 and 74 years at ten centers in order to determine the effectiveness of screening to reduce cancer mortality. Half of the participants were randomized into the screening arm and half into the control arm. Participants in the screening arm received annual screens for the four cancers for the first 6 years; participants in the control arm received usual care. Enrollment began in 1993 and concluded in 2001. Both arms were followed for cancer incidence and mortality for at least 13 years from baseline. Details of this study have been previously described and are available online (http://dcp.cancer.gov/plco). In 2006, FFPE pathology tissue samples were collected from PLCO participants who developed selected cancers, including colorectal cancer.

Women's Health Initiative (WHI)[16]

The WHI study is a large, multi-center study of postmenopausal women aged 50 to 79 years at recruitment from 40 US clinical centers between 1993 and 1998, including over 68,000

women who participated in four overlapping trials evaluating: menopausal hormone therapy (HT: two trials), dietary modification (DM) and calcium-vitamin D (CaD) supplementation. Participants in the CaD trial were recruited from those who were either in the HT or the DM trial. Details of the WHI study design have been described elsewhere and are available online (https://www.whi.org/). FFPE pathology tissue samples were collected from WHI participants who developed selected cancers, including colorectal cancer. Patients with sufficient material and consent were included in this study.

Whole Exome Sequencing

The training dataset of whole-exome sequenced (WES) samples were processed as described previously[17]. Formalin-fixed paraffin embedded (FFPE) tissues from CRCs were macrodissected and DNA extracted using the QIAamp DNA FFPE Tissue kit (Qiagen, Hilden, Germany) using standard protocols. Peripheral blood-derived DNAs were extracted using DNeasy blood and tissue kit (Qiagen) and sequenced as germline references. Capture of the whole exome was performed using Agilent Clinical Research Exome V2 (Agilent, Santa Clara, CA) with sequencing performed on an Illumina NovaSeq 6000 (San Diego, CA) comprising 150bp paired-end reads at the Australian Genome Research Facility.

Mean on-target coverage across *MUTYH* was 581.2 ± 156.9 (mean ± SD) for the tumor DNA samples and 372.0 ± 148.3 for blood-derived DNA samples.

Targeted Sequencing

The panel-sequenced tumors were processed as described previously[18]. Tumor DNA was extracted from FFPE sections and matching normal DNA from the blood, buccal, saliva, or adjacent normal colonic FFPE tissues was isolated. Tumor tissue was macrodissected from slides guided by a H&E stained slide marked for the tumor regions. All tumors underwent a

pathology review to confirm that the tumor was a primary colorectal carcinoma. DNA was extracted from FFPE tissue using the QIAamp DNA Mini or QIAamp DNA FFPE tissue kits and normal DNA from other tissues using standard DNA extraction methods. DNA concentrations were determined by Quant-iT PicoGreen dsDNA Assay or the Qubit dsDNA HS Assay kits.

DNA extracted from FFPE tissues was subjected to repair by using the PreCR Repair Mix (New England BioLabs, Ipswich, MA). AmpliSeq target amplification was performed using 20 ng of genomic DNA for each of the 2 AmpliSeq primer pools. Following removal of primers, PCR products from each pool were combined and subjected to end repair and A-tailing using the KAPA HyperPrep Kit (Roche). Adapter ligation was performed using the NEXTflex DNA barcodes Kit (PerkinElmer) and libraries were analyzed on High Sensitivity TapeStation and submitted for cluster generation. Barcoded DNA sequence libraries were pooled using 48 samples for tumors and 48 or 192 samples for normal DNA. Paired-end sequencing was performed on HiSeq 2500 using the Illumina Genome Analyzer operating procedure. Paired-end reads were aligned to the reference human genome (GRCh37/hg19) using Burrows-Wheeler Aligner (BWA-MEM version 0.7.9a). Local realignments and base quality recalibrations were performed on aligned data. Only reads aligned uniquely to the reference human GRCh37/hg19 genome assembly were used in downstream analysis.

Bioinformatics Pipelines and Analysis

WES samples were aligned to the GRCh37 human reference genome using BWA 0.7.12, from FASTQ files trimmed with trimmomatic 0.38[19] to remove adapter sequences. Somatic single-nucleotide variants (SNVs) and short insertions and deletions were called with Strelka

2.9.2[20] and Mutect2[21]. PASS variants that were reported by both callers were retained, and further filtered to those with a variant allele fraction ≥ 0.1, and a tumor depth ≥ 25 bases.

Somatic variants were generated from the panel-sequenced tumors as described previously[18]. Somatic SNVs were called using Strelka v1.0.1547 and MuTect v1.1.748, retaining only variants reported by both callers. Additional filters were applied based on strand bias, minor allele frequency in Exome Aggregation Consortium (ExAC), read-depth, alternative read-depth, and clustered read position[18]. Amplicon artifact filtering was applied to remove cases where mutant allele frequency varied across read clusters. Indel calls were obtained using majority votes from VarScan2 v2.4.349[22], VarDict (Feb 2017)[23], and Strelka v1.0.1547. After initial filtering of indels based on coverage and mutant allele frequency, background signals of alternative reads in normal samples were identified. A background filter was constructed from read counts from tumors and normal samples to remove indel calls in a subset of samples where signals were not significantly higher than background.

Germline variant calls were generated across the panel-sequenced samples using Strelka 2.9.2[20] following Illumina's recommended workflow. All called variants were annotated with CADD v1.6[24], REVEL[25,26], gnomAD v2.0.2[27] and ClinVar v20191219[28] so that they may be assessed and filtered based on population frequency and likelihood of pathogenicity.

Loss of heterozygosity (LOH) across all cohorts was assessed using the LOH calculation tool LOHdeTerminator v0.5[29], which determines likely regions of LOH by identifying heterozygous germline variants with somatic equivalents skewed towards homozygosity. With the lower mutation count across panel-sequenced data, we additionally required at least one somatic variant suggestive of LOH to occur within 100,000 bases of *MUTYH*.

We assessed the prevalence of copy number loss across *MUTYH* in CRCs with publicly available data from Pan-Cancer Analysis of Whole Genomes (PCAWG)[30] and The Cancer

Genome Atlas (TCGA)[31]. For PCAWG, copy number loss was considered to be any segment spanning *MUTYH* with predicted copy number less than two. For TCGA, loss was considered to be any segment spanning *MUTYH* with mean $\log_2$(copy-number/2) less than -0.3[32].

Microsatellite instability (MSI) status was determined using the method described by MSIseq v1.0[33], such that tumors observed to have a MSIseq threshold >1.9 were considered MSI-high.

Mutational signatures were calculated using the simulated annealing method previously described by SignatureEstimation[34]. We used the implementation of simulated annealing from the Python (v3.7.4) SciPy (v1.4.1)[35] optimization library ("basinhopping") with the L-BFGS-B optimization algorithm[36,37] to calculate the linear combination of TMSs that minimized reconstruction error, using a restricted set of 14 signatures that have previously been observed in the Pan-Cancer Analysis of Whole Genomes (PCAWG) cohort of 59 whole-genome sequenced CRC tumors[38], including the known base excision repair signatures SBS18 and SBS36 associated with defective *MUTYH*[39] and SBS30 associated with defective *NTHL1*[17,40], taken from the pre-defined set of COSMIC version 3.1 single base substitution (SBS) signatures[41].

The reconstruction error was calculated as the cosine distance between the observed mutational context counts and the predicted mutational context counts as computed from the calculated mutational signatures, a value bounded by 0 and 1, 0 indicating maximal similarity.[42]

Variant Classification

All variants detected within *MUTYH*, both somatic and germline, were classified into three categories:

Pathogenic: a variant annotated as pathogenic or likely pathogenic in ClinVar[28];

Potentially pathogenic or uncertain: a variant that has not been annotated as pathogenic in ClinVar, but has a gnomAD frequency of less than 1%, *and* exhibits any of the following:

- ClinVar classification as either variant of uncertain clinical significance or with conflicting interpretations of pathogenicity (VUS);
- Computational prediction of pathogenicity: REVEL>0.6[25]; or
- Computational prediction of pathogenicity: CADD>20[24].

Not pathogenic: all other variants.

Each tumor was classified based on these variant categories.

Determining a confidence threshold for TMSs

The training dataset consisted of 102 whole-exome sequenced CRCs, of which eight were known *MUTYH* positives and 92 were known *MUTYH* negatives. To establish a panel-specific threshold, we simulated panel-based results by restricting variant calls to the capture region of the panel, then calculating mutational signatures on this reduced set of variant calls. We fit a beta distribution to the sum of SBS18 and SBS36 to calculate confidence intervals[17] and selected 95% confidence as the cutoff for the classifier (Supplementary Table 9; Supplementary Figure 4).

FIGURES

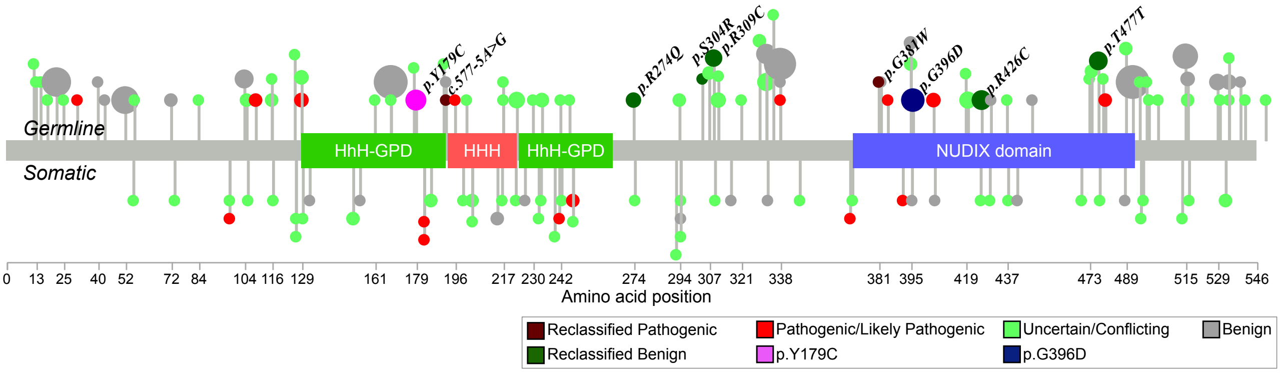

Supplementary Figure 1: Germline and somatic variants identified in the *MUTYH* (NM_001128425.1) gene relative to its functional domains observed across the 5,649 CRCs from the training, validation and test datasets. Variants are colored by pathogenicity as indicated by the ClinVar database, which includes the two most common pathogenic variants (p.Y179C and p.G396D), and variants reclassified by our SBS18/SBS36 TMS algorithm. The number of times a variant was observed is indicated by the radius of the data point. Source data are provided as a Source Data file.

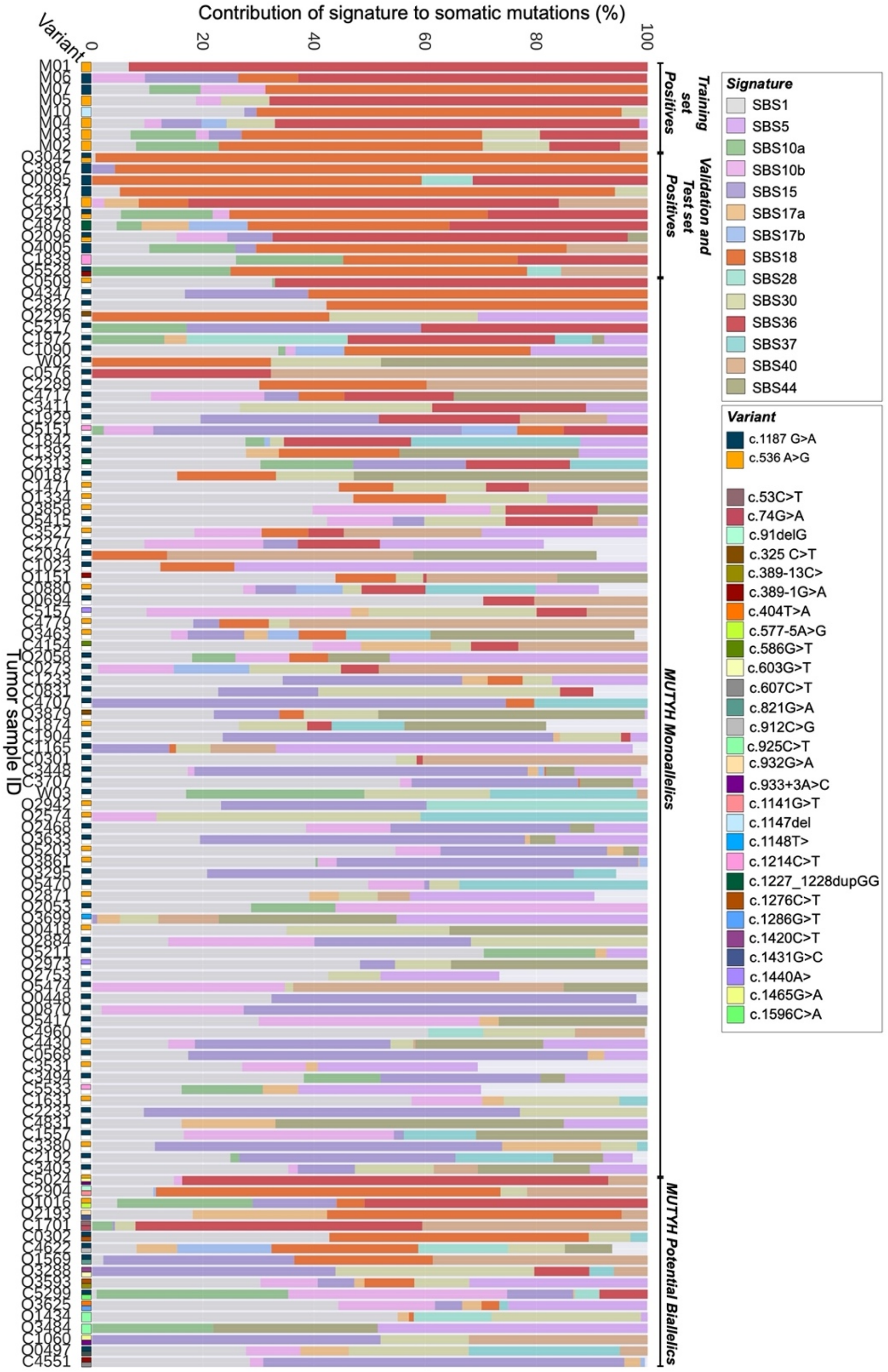

Supplementary Figure 2: Observed CRC TMS profiles derived from 14 COSMIC signatures relevant to CRC. Shown are the 19 CRCs from germline biallelic *MUTYH* pathogenic variant carriers (*MUTYH* positives), the 79 CRCs from germline monoallelic *MUTYH* pathogenic variant carriers (*MUTYH* monoallelics) and the 17 CRCs from the potential *MUTYH* biallelic group. All the CRCs from the germline biallelic *MUTYH* pathogenic variant carriers exhibit dominant SBS18 and/or SBS36 TMS. Source data are provided as a Source Data file.

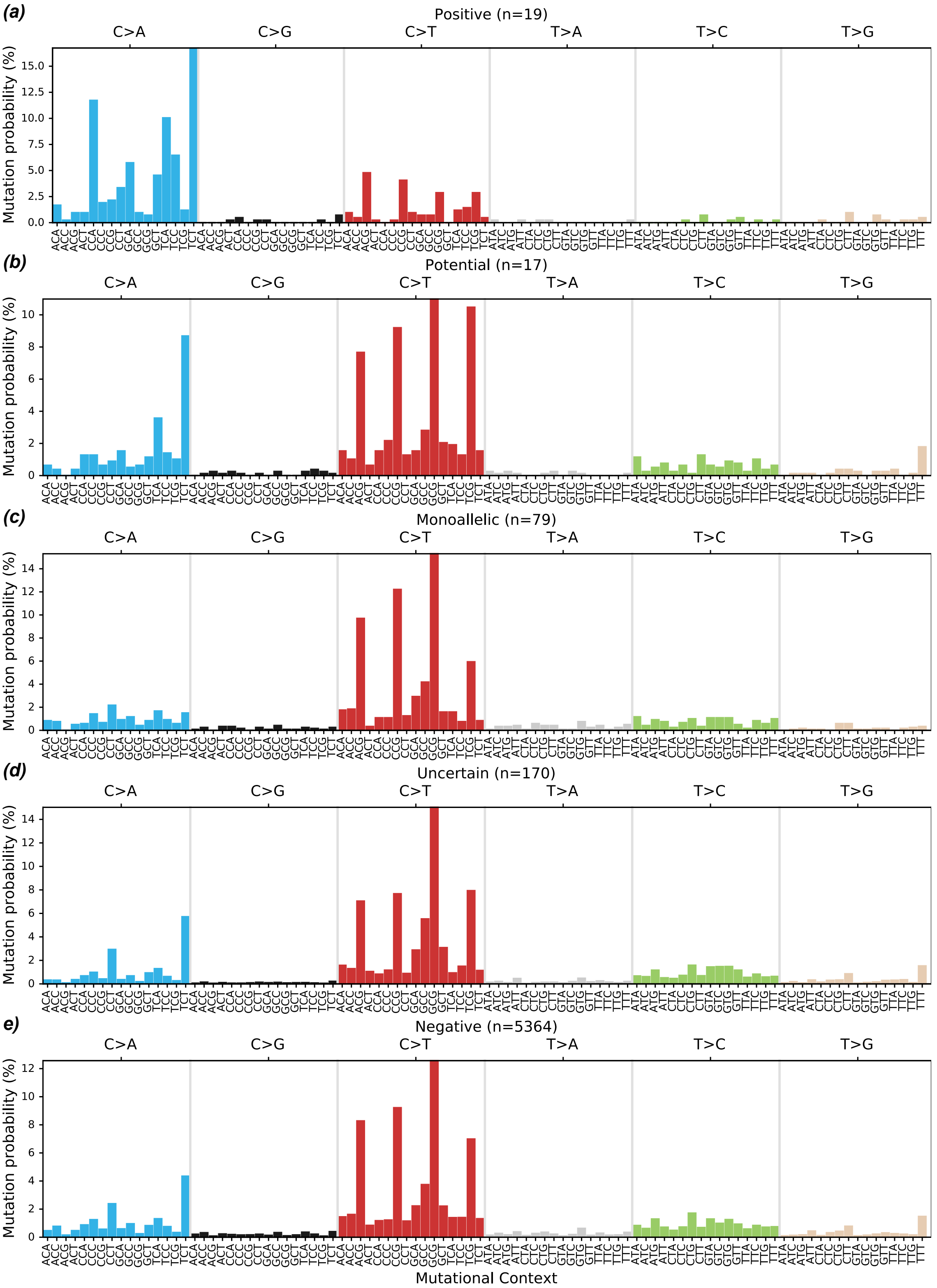

Supplementary Figure 3: 96-context proportions generated from the aggregate of all somatic mutations for (a) *MUTYH* positives, (b) potential *MUTYH* biallelics, (c) *MUTYH* monoallelics, (d) *MUTYH* uncertain and (e) *MUTYH* negatives. Source data are provided as a Source Data file.

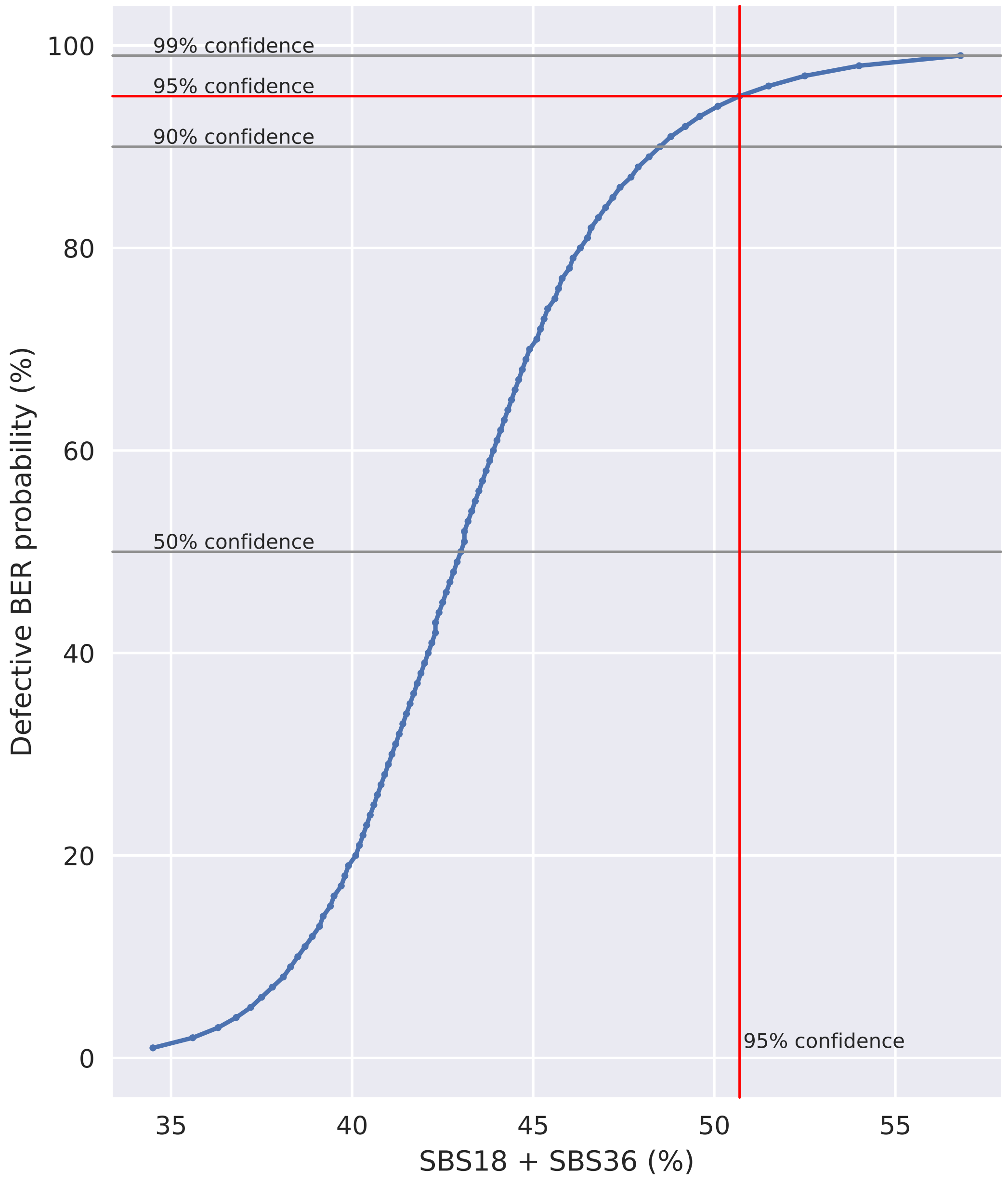

Supplementary Figure 4: Probability of a CRC tumor harboring biallelic *MUTYH* pathogenic variants given the sum of SBS18 and SBS36 TMS. A SBS18/36 TMS of >51% confers a >95% probability that the CRC tumor arose from base excision repair (BER) deficiency caused by germline biallelic pathogenic variants in the *MUTYH* gene. Source data are provided as a Source Data file.

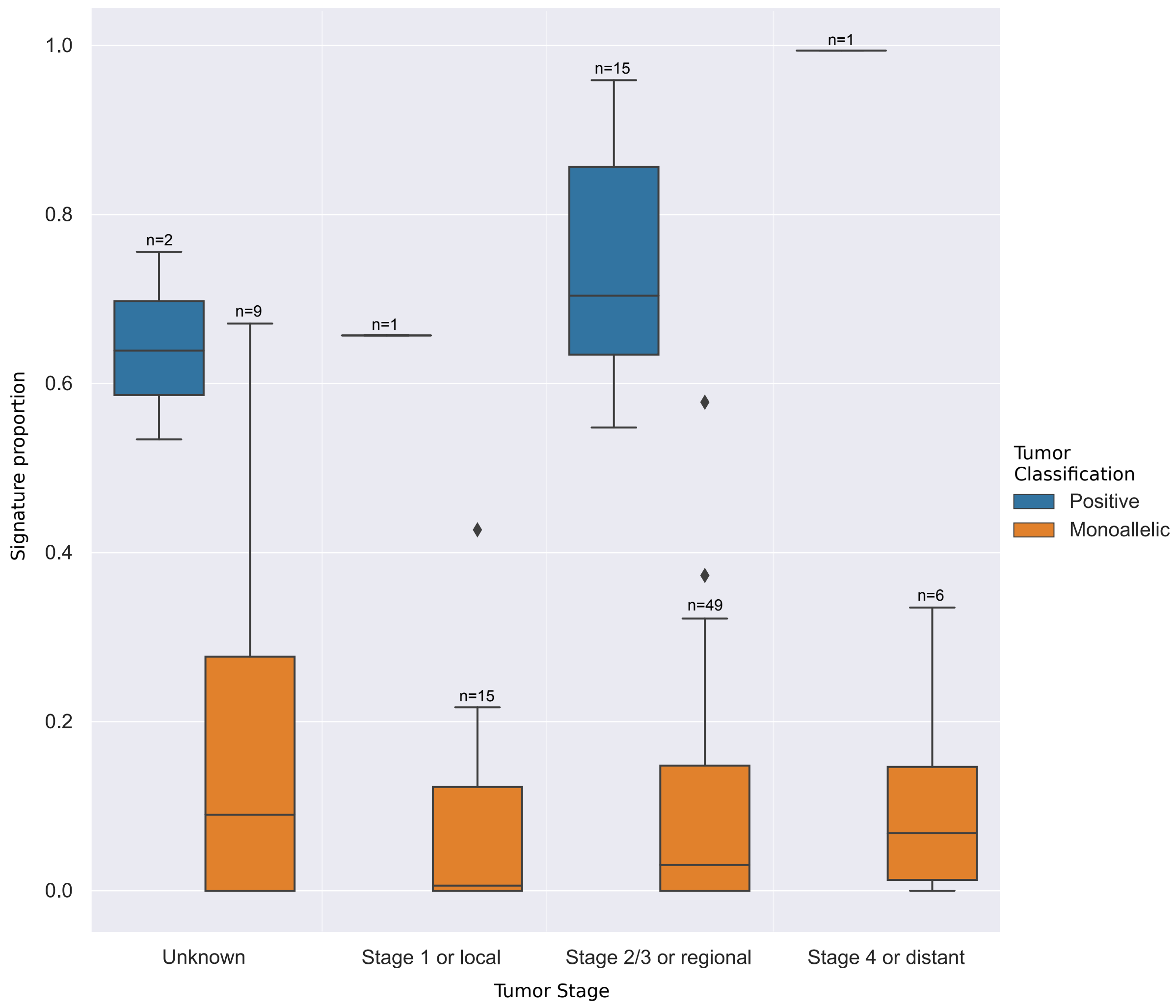

Supplementary Figure 5: Distribution of SBS18+SBS36 in 98 CRCs identified as *MUTYH* biallelic carriers (positives; n=19) and monoallelic carriers (n=79), stratified by tumor stage. All boxes correspond to the 25th and 75th percentiles and the whiskers represent 1.5x the inter-quartile range (IQR) extending from the boxes. Lines at the middle of each box show the median. Individual observations are shown beyond the whiskers. Source data are provided as a Source Data file.

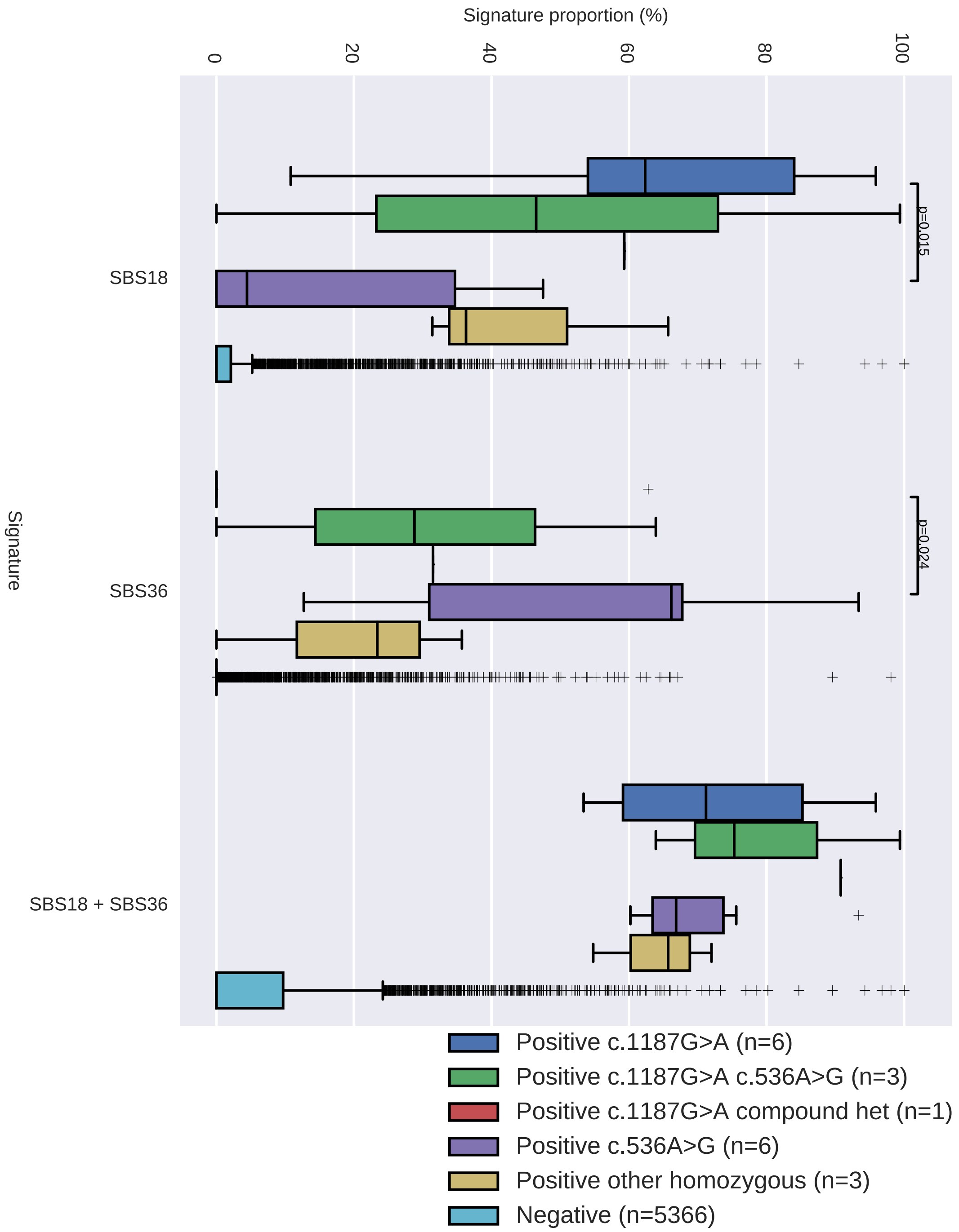

Supplementary Figure 6: Association of SBS18/SBS36 TMS with *MUTYH* germline pathogenic variant type. The CRCs from biallelic *MUTYH* carriers support the hypothesis that SBS36 is associated with c.536A>G p.Y179C, while SBS18 is associated with c.1187G>A p.G396D. P-values were calculated by applying a two-sided t-test comparing homozygous tumors. All boxes correspond to the 25th and 75th percentiles and the whiskers represent 1.5x the inter-quartile range (IQR) extending from the boxes. Lines at the middle of each box show the median. Individual observations are shown beyond the whiskers. Source data are provided as a Source Data file.

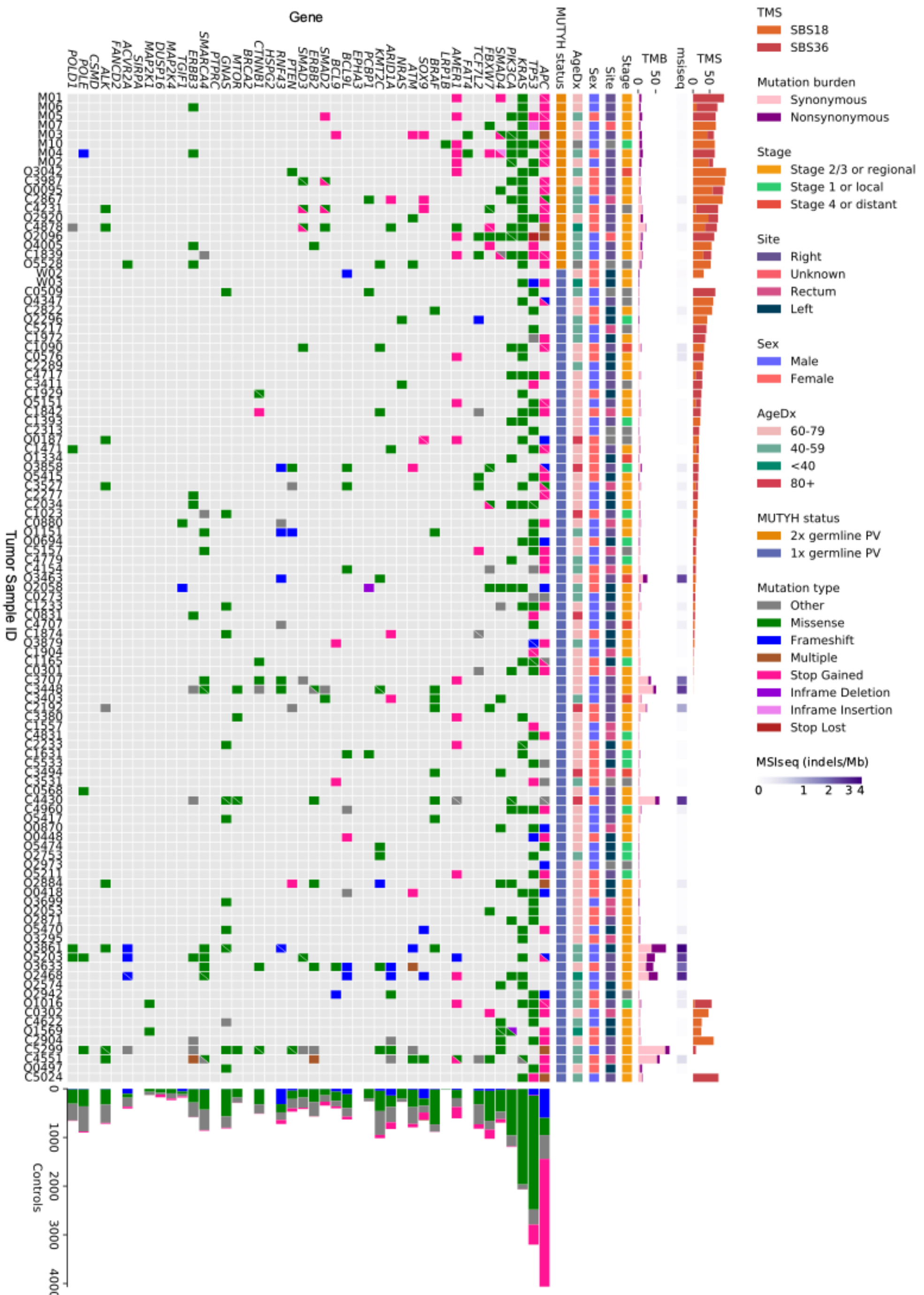

Supplementary Figure 7: Somatic mutation landscape of CRC tumors with biallelic or monoallelic *MUTYH* status. The 40 most commonly mutated CRC genes[43] are included, as well as known CRC genes *ALK*, *CSMD1*, *POLE*, and *POLD1*. *KRAS* was found to be significantly more commonly mutated in our biallelic *MUTYH* carriers. MSIseq[33] indicates tumors likely to be mismatch repair deficient and is measured as microindels in simple sequence repeats per megabase (Mb). Source data are provided as a Source Data file. AgeDx=age of diagnosis; TMB=tumour mutational burden (mutations/Mb); TMS=tumor mutational signature.

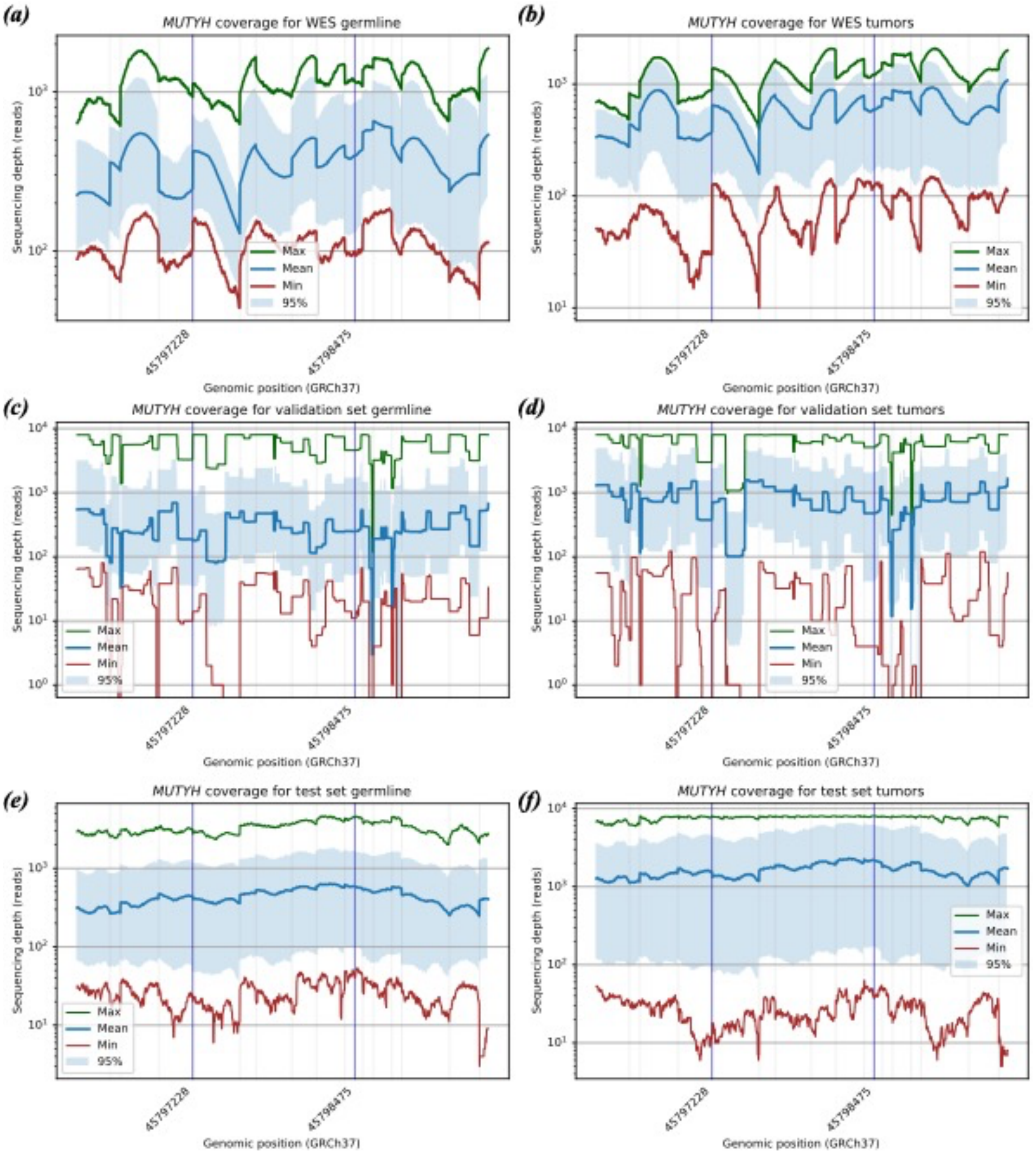

Supplementary Figure 8: Sequencing coverage across *MUTYH* for (a) whole exome sequenced (WES) training set germline, (b) WES training set tumors, (c) validation set germline, (d) validation set tumors, (e) test set germline and (f) test set tumors. Data are presented as mean (blue), maximum (green), minimum (red) and light blue extends from the 5th to 95th percentiles of observed sequencing depth at each exonic base in *MUTYH*. Source data are provided as a Source Data file.

TABLES

Supplementary Table 1: Each tumor was categorized into one of five groups, based on the type and number of variants identified in *MUTYH*. Only short variants (single nucleotide and indels) were considered when categorizing each tumor. PV=pathogenic variant; VUS=variant of uncertain significance.

**Groups**	**Germline pathogenic variant**	**Somatic pathogenic variant**	**Germline VUS**	**Somatic VUS**
***MUTYH* Positive**	2+ heterozygous<br>1+ homozygous	Any	Any	Any
***MUTYH* Monoallelic**	1 heterozygous	0	0	0
**Potential *MUTYH* biallelics**	2 or more heterozygous or 1 or more homozygous of any of these,<br>but *not* two heterozygous PVs or one homozygous PV			
***MUTYH* Uncertain**	0	1 heterozygous of any of these		
***MUTYH* Negative**	0	0	0	0

Supplementary Table 2: Studies included in the analysis. Each study and the total number of tumors included in each component of the analysis from that study are shown.

**Study**	**Study Total**	**Training Set**	**Validation Set**	**Test Set**	**% of total**
ACCFR[a]	356	47		309	6%
ANGELS	55	55			1%
CORSA	174		174		3%
CPSII	540		540		10%
CRA	327			327	6%
CRCGEN	314			314	6%
CRCGEN_2	335			335	6%
DACHS	278		278		5%
HCCS	108			108	2%
HPFS	273			273	5%
IWHS	377			377	7%
MCCS	413			413	7%
NHS	403			403	7%
NHSII	40			40	1%
OFCCR	679		679		12%
PLCO	120			120	2%
SCCFR	523		523		9%
WHI	334		334		6%
**Total**	**5649**	**102**	**2528**	**3019**	**100%**

[a] there was no overlap between CRC tumors from the ACCFR in the training and test sets

Supplementary Table 3: Distribution of clinical features of interest across each of the training, validation, and test sets. [1]Primary tumor location data were recorded based on International Classification of Disease (ICD) across studies and classified into right-sided, left-sided, rectal or unspecified. We classified caecum, ascending, hepatic flexure and transverse colon as right-sided. Splenic flexure, descending and sigmoid colon as left-sided, and rectosigmoid junction and rectum as rectal cancers. Source data are provided as a Source Data file.

	**Training set**	**Validation set**	**Test set**
Count	102	2,528	3,019
Age of diagnosis<br>mean ± sd (min-max)	42.7 ± 11.6<br>(25-71)	65.8 ± 12.3<br>(23-93)	66.4 ±11.8<br>(20-92)
**Sex**			
Male<br>Female	40 (39.2%)<br>62 (60.8%)	1,108 (43.8%)<br>1,420 (56.2%)	1,383 (45.8%)<br>1,636 (54.2%)
**Self-reported race**			
American Indian or Alaska Native	0 (0%)	3 (0.1%)	1 (0.03%)
Asian	4 (4%)	16 (0.6%)	22 (0.7%)
Black or African-American	1 (1%)	37 (1.5%)	21 (0.7%)
White	87 (85%)	2,408 (95.3%)	2,456 (81.4%)
Other	0 (0%)	30 (1.2%)	15 (0.5%)
Unspecified	10 (10%)	34 (1.3%)	504 (16.7%)
**Cancer Site[1]**			
Right-sided	46 (45.1%)	1,136 (44.9%)	1,190 (39.4%)
Left-sided	30 (29.4%)	754 (29.8%)	842 (27.9%)
Rectal	21 (20.6%)	586 (23.2%)	732 (24.2%)
Unspecified/Other	5 (4.9%)	52 (2.1%)	255 (8.4%)
**Stage**			
Stage 0	0 (0%)	2 (0.07%)	0 (0%)
Stage 1	11 (10.8%)	554 (21.9%)	509 (16.9%)
Stage 2/3	63 (61.8%)	1,404 (55.5%)	1,592 (52.7%)
Stage 4	18 (17.6)	193 (7.6%)	332 (11.0%)
Unspecified	10 (9.8%)	375 (14.8%)	586 (19.4%)

Supplementary Table 4: Distribution of participant characteristics and clinic-pathological features across each of the tumor categories. Source data are provided as a Source Data file.

	***MUTYH* Positive**	***MUTYH* Negative**	***MUTYH* Monoallelic**	**Potential *MUTYH* biallelics**	***MUTYH* Uncertain**
Count	19	5,364	79	17	170
Age of diagnosis mean ± sd (min-max)	53.7 ± 10.1 (33-67)	65.8 ± 12.4 (20-93)	65.6 ± 11.4 (35-91)	57.2 ± 17.0 (33-89)	64.6 ± 13.2 (32-88)
**Sex**					
Male<br>Female	10 (52.6%)<br>9 (47.4%)	2,396 (44.7%)<br>2,968 (55.3%)	41 (51.9%)<br>38 (48.1%)	8 (47.1%)<br>9 (52.9%)	76 (44.7%)<br>94 (55.3%)
**Self-reported race**					
American Indian or Alaska Native	0 (0%)	4 (0.07%)	0 (0%)	0 (0%)	0 (0%)
Asian	0 (0%)	38 (0.7%)	0 (0%)	1 (5.9%)	3 (1.8%)
Black or African-American	0 (0%)	57 (1.1%)	0 (0%)	0 (0%)	2 (1.2%)
White	18 (94.7%)	4,708 (87.8%)	70 (88.6%)	13 (76.5%)	145 (85.3%)
Other	0 (0%)	40 (0.7%)	1 (1.3%)	0 (0%)	4 (2.4%)
Unspecified	1 (5.3%)	517 (9.6%)	8 (10.1%)	3 (17.6%)	16 (9.4%)
**Cancer Site**					
Right-sided	16 (84.2%)	2,243 (41.8%)	29 (36.7%)	6 (35.3%)	81 (47.6%)
Left-sided	0 (0%)	1,556 (29.0%)	24 (30.4%)	6 (35.3%)	40 (23.5%)
Rectal	1 (5.3%)	1,272 (23.7%)	21 (26.6%)	5 (29.4%)	40 (23.5%)
Unspecified/Other	2 (10.5%)	293 (5.5%)	5 (6.3%)	0 (0%)	9 (5.3%)
**Stage**					
Stage 0	0 (0%)	2 (0.04%)	0 (0%)	0 (0%)	0 (0%)
Stage 1	1 (5.3%)	1,028 (19.2%)	14 (17.7%)	3 (17.6%)	28 (16.5%)
Stage 2/3	14 (73.7%)	2,891 (53.9%)	46 (58.2%)	12 (70.6%)	101 (59.4%)
Stage 4	1 (5.3%)	522 (9.7%)	6 (7.6%)	1 (5.9%)	13 (7.6%)
Unspecified	3 (15.8%)	921 (17.2%)	13 (16.5%)	1 (5.9%)	28 (16.5%)

Supplementary Table 5: Distribution of clinical features across each of the included studies. Site categories are R=Right-sided, L=Left-sided, Rct=Rectal, Un=Unspecified. Stage categories are 0=Stage 0, 1=Stage 1, 23=Stage 2/3, 4=Stage 4, Un=Unspecified. Source data are provided as a Source Data file.

**Study**	**Age of diagnosis**	**Sex (female)**	**Site % R/L/Rct/Un**	**Stage % 0/1/23/4/Un**
ACCFR	46.9 ± 8.5 (20-71)	170 (47.8%)	28/30/37/4	0/12/74/8/6
ANGELS	37.2 ± 8.7 (25-63)	35 (63.6%)	35/38/27/0	0/11/58/25/5
CORSA	66.6 ± 11.3 (34-91)	67 (38.5%)	32/31/31/6	0/9/34/13/43
CPSII	74.5 ± 6.8 (56-90)	288 (53.3%)	53/27/20/0.1	0/43/53/5/0
CRA	65.9 ± 11.5 (25-92)	141 (43.1%)	45/24/31/0	0/16/50/10/23
CRCGEN	68.4 ± 10.9 (31-91)	114 (36.3%)	15/23/23/40	0/10/39/11/40
CRCGEN_2	67.3 ± 10.6 (22-90)	129 (38.5%)	33/40/16/10	0/7/71/11/11
DACHS	69.7 ± 10.4 (37-91)	123 (44.2%)	39/27/33/0	0/19/69/12/0.3
HCCS	58.5 ± 11.6 (21-86)	42 (38.9%)	16/21/18/45	0/19/18/7/56
HPFS	71.6 ± 8.9 (47-92)	0 (0%)	46/25/21/8	0/23/52/12/13
IWHS	74.5 ± 5.5 (58-85)	377 (100%)	57/26/16/11	0/31/42/12/15
MCCS	69.1 ± 7.9 (44-85)	191 (46.2%)	37/28/33/19	0/8/37/16/39
NHS	68.7 ± 8.3 (42-86)	403 (100%)	53/29/18/2	0/22/65/10/3
NHSII	54.6 ± 5.8 (39-64)	40 (100%)	38/35/28/0	0/20/68/8/5
OFCCR	57.5 ± 10.0 (28-82)	337 (49.8%)	33/36/28/3	0/18/67/10/5
PLCO	69.3 ± 6.4 (55-85)	56 (46.7%)	62/20/17/2	0/30/63/6/0.8
SCCFR	58.3 ± 11.0 (23-74)	271 (51.6%)	44/33/20/3	0.3/11/35/3/50
WHI	76.5 ± 7.3 (58-93)	334 (100%)	70/18/10/2	0/22/68/8/2

Supplementary Table 6: Biallelic *MUTYH* carriers CRCs (*MUTYH* positives) and their clinicopathological characteristics. Homozygous pathogenic variants are indicated as (H). AgeDx=age of diagnosis.

**ID**	**Cohort**	**Pathogenic Variant(s)**	**Sex**	**Age Dx**	**SBS18 (%)**	**SBS36 (%)**
M01[a]	WES	c.536A>G p.Y179C (H)	Male	60-69	0	93
M02[a]	WES	c.536A>G p.Y179C (H)	Male	60-69	48	13
M03[a]	WES	c.536A>G p.Y179C (H)	Male	60-69	43	19
M04[b]	WES	c.536A>G p.Y179C (H)	Female	50-59	0	66
M05[b]	WES	c.536A>G p.Y179C (H)	Female	50-59	0	68
M06[c]	WES	c.1187G>A p.G396D (H)	Male	60-69	11	63
M07[c]	WES	c.1187G>A p.G396D (H)	Male	60-69	69	0
M10	WES	c.1147del p.A385PfsTer23 (H)	Male	30-39	66	0
O0095	Validation	c.1187G>A p.G396D (H)	Female	40-49	59	32
O2096	Validation	c.1187G>A p.G396D<br>c.536A>G p.Y179C	Male	50-59	0	64
O2920	Validation	c.1187G>A p.G396D<br>c.536A>G p.Y179C	Female	60-69	47	29
O3042	Validation	c.1187G>A p.G396D<br>c.536A>G p.Y179C	Female	40-49	99	0
O4005	Validation	c.1187G>A p.G396D (H)	Male	50-59	56	0
O5528	Validation	c.1187G>A p.G396D<br>c.389-1G>A	Female	NA	53	0
C1839	Test	c.1214C>T p.P405L (H)	Male	40-49	31	23
C2867	Test	c.1187G>A p.G396D (H)	Male	50-59	89	0
C3987	Test	c.1187G>A p.G396D (H)	Female	60-69	96	0
C4231	Test	c.536A>G p.Y179C (H)	Female	40-49	9	67
C4878	Test	c.1227_1228dupGG<br>p.E410Gfs*43 (H)	Female	30-39	36	36

[a, b, c] represent three different individuals who presented with synchronous CRCs that each underwent WES.

Supplementary Table 7: The number of CRCs and the mean ± standard deviation for their combined SBS18/36 value, Mutational signature reconstruction error and somatic mutation count for each of the five tumor classes defined in this study by training, validation and test datasets. Source data are provided as a Source Data file.

Tumor Class/Cohort	n	SBS18/36 (%)	Reconstruction Error (%)	Somatic Mutations
***MUTYH* Positive**	**19**	**72.9 ± 14.4**	**19.2 ± 8.7**	**22.0 ± 8.8**
Training	8	69.8 ± 10.4	18.3 ± 8.7	21.9 ± 9.0
Validation	6	73.1 ± 18.9	21.7 ± 8.1	19.3 ± 6.5
Test	5	77.5 ± 16.0	17.5 ± 10.5	25.4 ± 11.5
**Potential *MUTYH* biallelics**	**17**	**25.2 ± 26.5**	**29.9 ± 17.6**	**46.1 ± 71.4**
Training	0	n/a	n/a	n/a
Validation	9	17.4 ± 22.4	32.0 ± 17.4	46.3 ± 80.7
Test	8	34.0 ± 29.5	27.5 ± 18.8	45.8 ± 64.8
***MUTYH* Monoallelic**	**79**	**10.8 ± 15.4**	**37.0 ± 17.8**	**15.1 ± 21.6**
Training	2	16.1 ± 22.8	55.4 ± 9.8	5.5 ± 0.7
Validation	32	7.3 ± 13.8	34.3 ± 18.9	14.6 ± 19.5
Test	45	13.1 ± 16.1	38.1 ± 16.9	16.0 ± 23.5
***MUTYH* Uncertain**	**170**	**7.9 ± 14.4**	**27.5 ± 19.0**	**60.4 ± 122.4**
Training	0	n/a	n/a	n/a
Validation	57	9.1 ± 16.3	32.3 ± 21.7	44.1 ± 79.5
Test	113	7.4 ± 13.3	25.2 ± 17.0	68.7 ± 138.7
***MUTYH* Negative**	**5,364**	**7.1 ± 12.4**	**33.6 ± 17.6**	**21.8 ± 46.4**
Training	92	4.4 ± 8.3	30.2 ± 20.7	29.3 ± 40.0
Validation	2,424	7.4 ± 13.2	34.8 ± 18.7	20.9 ± 44.0
Test	2,848	6.9 ± 11.7	32.6 ± 16.3	22.2 ± 48.4
**Total**	**5,649**	**7.4 ± 13.2**	**33.4 ± 17.6**	**22.9 ± 50.6**
Training	102	9.7 ± 19.7	29.7 ± 20.4	28.3 ± 38.2
Validation	2,528	7.6 ± 13.8	34.7 ± 18.7	21.5 ± 45.2
Test	3,019	7.2 ± 12.4	32.4 ± 16.5	23.9 ± 55.0

Supplementary Table 8: Tumor mutational signatures calculated from the aggregate of all somatic mutations for each class. Source data are provided as a Source Data file.

	Positive	Monoallelic	Negative	Potential	Uncertain
**n**	19	79	5,364	17	170
**Mutations**	418	1,196	116,670	783	10,274
**SBS1**	10.4%	30.5%	24.3%	23.4%	17.8%
**SBS5**	0%	28.5%	29.1%	16.6%	18.7%
**SBS10a**	1.4%	0%	3.5%	6.3%	6.1%
**SBS10b**	1.7%	3.1%	7.9%	15.1%	11.1%
**SBS15**	0.4%	27.0%	22.6%	16.3%	35.1%
**SBS17a**	0.6%	0.2%	1.0%	2.0%	1.3%
**SBS17b**	1.1%	0.2%	0.9%	0.1%	1.0%
**SBS18**	35.8%	6.0%	2.9%	5.0%	0%
**SBS28**	0%	0%	0.1%	0%	0%
**SBS30**	0%	0.3%	0.6%	4.3%	0%
**SBS36**	48.5%	0.9%	2.2%	10.8%	0.8%
**SBS37**	0%	0%	0%	0%	0%
**SBS40**	0%	0%	0%	0%	0%
**SBS44**	0%	3.3%	4.8%	0%	8.1%
**Error**	4.1%	4.3%	2.7%	2.8%	2.2%

Supplementary Table 9: Probability of a tumor showing the *MUTYH*-related base excision repair deficiency tumor mutational signature (TMS) given an observed level of SBS18 + SBS36. Based on the training set of 102 CRCs and assuming a beta-distribution in the mutational signatures, a tumor can be classified as *MUTYH* positive with 95% confidence when SBS18 + SBS36 exceeds 51%. Source data are provided as a Source Data file.

**Base excision repair deficiency TMS probability**	**SBS18/36 (%)**
1%	35%
5%	37%
10%	39%
20%	40%
30%	41%
40%	42%
50%	43%
60%	44%
70%	45%
80%	46%
90%	49%
**95%**	**51%**
99%	57%

Supplementary Table 10: Distribution of somatic mutations, SBS18, SBS36, and SBS18+SBS36, for monoallelic and biallelic *MUTYH* carriers, stratified by tumor stage. Source data are provided as a Source Data file.

**Stage**	**n**	**Mutations (mean ± sd)**	**SBS18 (mean ± sd)**	**SBS36 (mean ± sd)**	**SBS18+SBS36 (mean ± sd)**
**Monoallelic Carriers (n=79)**					
Unknown	13	5.692 ± 2.496	0.061 ± 0.173	0.126 ± 0.210	0.186 ± 0.240
Stage 1 or Local	14	8.929 ± 4.446	0.063 ± 0.124	0.018 ± 0.049	0.081 ± 0.124
Stage 2/3 or regional	46	19.935 ± 27.053	0.043 ± 0.107	0.052 ± 0.093	0.095 ± 0.132
Stage 4 or distant	6	13.333 ± 6.563	0.106 ± 0.128	0.000 ± 0.000	0.106 ± 0.128
**Biallelic Carriers (n=19)**					
Unknown	3	22.333 ± 3.786	0.208 ± 0.286	0.435 ± 0.377	0.643 ± 0.111
Stage 1 or Local	1	23	0.657	0	0.657
Stage 2/3 or regional	14	22.643 ± 9.811	0.418 ± 0.314	0.315 ± 0.302	0.733 ± 0.139
Stage 4 or distant	1	11	0.994	0	0.994

Supplementary Table 11: Performance of individual hotspots for detecting *MUTYH* biallelic carriers. *KRAS* p.G12C detects 16 of the 19 positives, giving a sensitivity of 84.2% and specificity of 97.6%. Requiring both *KRAS* p.G12C and *PIK3CA* p.Q546K to be present increases the specificity to 99.98%, with only one false positive, while considering the presence of either hotspot to indicate a positive tumor detects 17 of 19 known positives. Source data are provided as a Source Data file.

	**Biomarker Present**	**Biomarker Absent**
**Prediction with *KRAS* p.G12C**		
Positive (n=19)	16 (84.2%)	3
Negative (n=5,364)	127	5,237 (97.6%)
**Prediction with *PIK3CA* p.Q546K**		
Positive (n=19)	7 (36.8%)	12
Negative (n=5,364)	36	5328 (99.3%)
**Prediction with both *KRAS* p.G12C and *PIK3CA* p.Q546K required**		
Positive (n=19)	6 (31.6%)	13
Negative (n=5,364)	1	5363 (99.98%)
**Prediction with either *KRAS* p.G12C or *PIK3CA* p.Q546K required**		
Positive (n=19)	17 (89.5%)	2
Negative (n=5,364)	162	5,202 (97.0%)

Supplementary Table 12: Pearson correlation comparing similarity between distribution of features of interest when 102 whole-exome sequenced (WES) tumors were down sampled from the 67Mb WES to the 1.96Mb and 1.34Mb panel captures. SBS18 and SBS36 alone exhibit moderate correlation, similar to SBS1 (a ubiquitous signature in cancer), but combining the two signatures substantially improved correlation, suggesting that combined signatures from panel data represent an accurate proxy for WES results. Source data are provided as a Source Data file.

**Feature**	**67 Mb WES (discovery) vs 1.34Mb (validation)**	**67Mb WES (discovery) vs 1.96Mb (test)**	**1.34Mb (validation) vs 1.96Mb (test)**
SBS18	0.554	0.500	0.900
SBS36	0.715	0.725	0.965
SBS18+SBS36	0.904	0.911	0.962
SBS1	0.570	0.559	0.884
Error	0.350	0.413	0.914
Mutations	0.954	0.974	0.992

Supplementary Table 13: Comparison of tumor mutational signatures calculated on somatic variants restricted to the whole-exome sequenced (WES) capture (67.3Mb), and the panel-based validation and test set captures (1.3Mb and 2.0Mb respectively). The sum of SBS18 and SBS36 (SBS18/36), somatic mutation count (mutations) and reconstruction error (error) are shown for the group of WES biallelic carriers (*MUTYH* positives) and non-carriers (*MUTYH* negatives) when applied to the different capture sizes. Although SBS18/36 remains consistent on average in the two groups across the different capture sizes, reconstruction error tends to be higher in the panel captures, due to the reduced number of variants available to calculate signatures. Source data are provided as a Source Data file. WES=whole exome sequenced.

**Group**	**Capture**	**SBS18/36 (%) Mean±SD (min-max)**	**Error (%) Mean±SD (min-max)**	**Mutations Mean±SD (min-max)**
***MUTYH* positives (n=8)**	WES 67.3Mb capture	67.7% ± 11.7% (48.0%-79.7%)	3.1% ± 0.9% (1.9%-4.2%)	694.4 ± 150.4 (446-914)
	Validation 1.3Mb capture	69.8% ± 10.4% (60.2%-93.4%)	18.3% ± 8.7% (8.9%-32.7%)	21.9 ± 9.0 (9-32)
	Test 2.0Mb capture	61.1% ± 7.5% (61.6%-82.8%)	17.8% ± 9.9% (4.7%-32.7%)	28.3 ± 9.2 (9-36)
***MUTYH* negatives (n=92)**	WES 67.3Mb capture	5.8% ± 6.9% (0.0%-39.0%)	7.9% ± 8.7% (0.1%-49.0%)	991.4 ± 1,914.5 (7-10,998)
	Validation 1.3Mb capture	4.4% ± 8.3% (0.0%-37.5%)	30.2% ± 20.7% (0.5%-78.3%)	29.3 ± 40.0 (2-235)
	Test 2.0Mb capture	4.9% ± 8.4% (0.0%-36.7%)	32.0% ± 20.4% (0.6%-80.9%)	30.3 ± 48.9 (3-280)

References

1. Newcomb, P. A. *et al.* Colon Cancer Family Registry: an international resource for studies of the genetic epidemiology of colon cancer. *Cancer Epidemiol. Biomarkers Prev.* **16,** 2331–2343 (2007).
2. Hofer, P. *et al.* MNS16A tandem repeats minisatellite of human telomerase gene: a risk factor for colorectal cancer. *Carcinogenesis* **32,** 866–871 (2011).
3. Calle, E. E. *et al.* The American Cancer Society Cancer Prevention Study II Nutrition Cohort: rationale, study design, and baseline characteristics. *Cancer* **94,** 2490–2501 (2002).
4. Campbell, P. T. *et al.* Establishment of the cancer prevention study II nutrition cohort colorectal tissue repository. *Cancer Epidemiol. Biomarkers Prev.* **23,** 2694–2702 (2014).
5. Cunningham, J. M. *et al.* The frequency of hereditary defective mismatch repair in a prospective series of unselected colorectal carcinomas. *Am. J. Hum. Genet.* **69,** 780–790 (2001).
6. Brenner, H. *et al.* Reduced risk of colorectal cancer up to 10 years after screening, surveillance, or diagnostic colonoscopy. *Gastroenterology* **146,** 709–717 (2014).
7. Jia, M. *et al.* No association of CpG island methylator phenotype and colorectal cancer survival: population-based study. *Br. J. Cancer* **115,** 1359–1366 (2016).
8. Giovannucci, E., Liu, Y., Platz, E. A., Stampfer, M. J. & Willett, W. C. Risk factors for prostate cancer incidence and progression in the health professionals follow-up study. *Int. J. Cancer* **121,** 1571–1578 (2007).
9. Haruki, K. *et al.* An integrated analysis of lymphocytic reaction, tumour molecular characteristics and patient survival in colorectal cancer. *Br. J. Cancer* **122,** 1367–1377 (2020).
10. Folsom, A. R. *et al.* Increased incidence of carcinoma of the breast associated with abdominal adiposity in postmenopausal women. *Am. J. Epidemiol.* **131,** 794–803 (1990).
11. Tillmans, L. S. *et al.* Associations between cigarette smoking, hormone therapy, and folate intake with incident colorectal cancer by TP53 protein expression level in a population-based cohort of older women. *Cancer Epidemiol. Biomarkers Prev.* **23,** 350–

355 (2014).

12. Giles, G. G. & English, D. R. The Melbourne Collaborative Cohort Study. *IARC Sci Publ* **156,** 69–70 (2002).
13. Belanger, C. F., Hennekens, C. H., Rosner, B. & Speizer, F. E. The nurses' health study. *Am. J. Nurs.* **78,** 1039–1040 (1978).
14. Bao, Y. *et al.* Origin, methods, and evolution of the three nurses' health studies. *Am. J. Public Health* **106,** 1573–1581 (2016).
15. Zhu, C. S. *et al.* The prostate, lung, colorectal and ovarian cancer (PLCO) screening trial pathology tissue resource. *Cancer Epidemiol. Biomarkers Prev.* **25,** 1635–1642 (2016).
16. Hays, J. *et al.* The Women's Health Initiative recruitment methods and results. *Ann Epidemiol* **13,** S18-77 (2003).
17. Georgeson, P. *et al.* Evaluating the utility of tumour mutational signatures for identifying hereditary colorectal cancer and polyposis syndrome carriers. *Gut* (2021). doi:10.1136/gutjnl-2019-320462
18. Zaidi, S. H. *et al.* Landscape of somatic single nucleotide variants and indels in colorectal cancer and impact on survival. *Nat. Commun.* **11,** 3644 (2020).
19. Bolger, A. M., Lohse, M. & Usadel, B. Trimmomatic: a flexible trimmer for Illumina sequence data. *Bioinformatics* **30,** 2114–2120 (2014).
20. Kim, S. *et al.* Strelka2: fast and accurate calling of germline and somatic variants. *Nat. Methods* **15,** 591–594 (2018).
21. McKenna, A. *et al.* The Genome Analysis Toolkit: a MapReduce framework for analyzing next-generation DNA sequencing data. *Genome Res.* **20,** 1297–1303 (2010).
22. Koboldt, D. C. *et al.* VarScan: variant detection in massively parallel sequencing of individual and pooled samples. *Bioinformatics* **25,** 2283–2285 (2009).
23. Lai, Z. *et al.* VarDict: a novel and versatile variant caller for next-generation sequencing in cancer research. *Nucleic Acids Res.* **44,** e108 (2016).
24. Rentzsch, P., Witten, D., Cooper, G. M., Shendure, J. & Kircher, M. CADD: predicting the deleteriousness of variants throughout the human genome. *Nucleic Acids Res.* **47,** D886–D894 (2019).

25. Ioannidis, N. M. *et al.* REVEL: an ensemble method for predicting the pathogenicity of rare missense variants. *Am. J. Hum. Genet.* **99,** 877–885 (2016).

26. Ioannidis, N. M. REVEL database. *REVEL: Rare Exome Variant Ensemble Learner - Downloads* at <https://sites.google.com/site/revelgenomics/downloads>

27. Karczewski, K. J. *et al.* The mutational constraint spectrum quantified from variation in 141,456 humans. *Nature* **581,** 434–443 (2020).

28. Landrum, M. J. *et al.* ClinVar: improving access to variant interpretations and supporting evidence. *Nucleic Acids Res.* **46,** D1062–D1067 (2018).

29. Georgeson, P. LOHdeTerminator. at <https://github.com/supernifty/LOHdeTerminator>

30. Dentro, S. C. *et al.* Characterizing genetic intra-tumor heterogeneity across 2,658 human cancer genomes. *Cell* **184,** 2239–2254.e39 (2021).

31. Cancer Genome Atlas Research Network *et al.* The Cancer Genome Atlas Pan-Cancer analysis project. *Nat. Genet.* **45,** 1113–1120 (2013).

32. NCI Genomic Data Commons. Bioinformatics Pipeline: Copy Number Variation Analysis - GDC Docs. at <https://docs.gdc.cancer.gov/Data/Bioinformatics_Pipelines/CNV_Pipeline/>

33. Huang, M. N. *et al.* MSIseq: Software for Assessing Microsatellite Instability from Catalogs of Somatic Mutations. *Sci. Rep.* **5,** 13321 (2015).

34. Huang, X., Wojtowicz, D. & Przytycka, T. M. Detecting presence of mutational signatures in cancer with confidence. *Bioinformatics* **34,** 330–337 (2018).

35. Virtanen, P. *et al.* SciPy 1.0: fundamental algorithms for scientific computing in Python. *Nat. Methods* **17,** 261–272 (2020).

36. Zhu, C., Byrd, R. H., Lu, P. & Nocedal, J. Algorithm 778: L-BFGS-B. *ACM Trans. Math. Softw.* **23,** 550–560 (1997).

37. Olson, B., Hashmi, I., Molloy, K. & Shehu, A. Basin hopping as a general and versatile optimization framework for the characterization of biological macromolecules. *Advances in Artificial Intelligence* **2012,** 1–19 (2012).

38. Alexandrov, L. B. *et al.* The repertoire of mutational signatures in human cancer. *Nature* **578,** 94–101 (2020).

39. Viel, A. *et al.* A Specific Mutational Signature Associated with DNA 8-Oxoguanine Persistence in MUTYH-defective Colorectal Cancer. *EBioMedicine* **20,** 39–49 (2017).

40. Grolleman, J. E., Díaz-Gay, M., Franch-Expósito, S., Castellví-Bel, S. & de Voer, R. M. Somatic mutational signatures in polyposis and colorectal cancer. *Mol. Aspects Med.* **69,** 62–72 (2019).

41. Wellcome Sanger Institute. COSMIC Signatures of Mutational Processes in Human Cancer. *Signatures of Mutational Processes in Human Cancer* (2019). at <https://cancer.sanger.ac.uk/cosmic/signatures>

42. Alexandrov, L. B., Nik-Zainal, S., Wedge, D. C., Campbell, P. J. & Stratton, M. R. Deciphering signatures of mutational processes operative in human cancer. *Cell Rep.* **3,** 246–259 (2013).

43. Martínez-Jiménez, F. *et al.* A compendium of mutational cancer driver genes. *Nat. Rev. Cancer* **20,** 555–572 (2020).

Supplementary Note 1 – Additional Acknowledgements

DDB was supported by a NHMRC Investigator grant (GNT1194896) and the University of Melbourne Dame Kate Campbell Fellowship. MAJ was supported by a NHMRC Investigator grant and the National Cancer Institute of the National Institutes of Health (grant U01CA167551). BJP was supported by a Victorian Health and Medical Research Fellowship.

Genetics and Epidemiology of Colorectal Cancer Consortium (GECCO): UP was supported by National Cancer Institute, National Institutes of Health, U.S. Department of Health and Human Services grants U01 CA137088 and U01 CA164930. LH was supported by grant R21 CA191312. ATC were supported by R01 CA176272. Genotyping/Sequencing services were provided by the Center for Inherited Disease Research (CIDR) contract numbers HHSN268201700006I and HHSN268201200008I. This research was funded in part through the NIH/NCI Cancer Center Support Grant P30 CA015704. Scientific Computing Infrastructure at Fred Hutch funded by ORIP grant S10OD028685.

Amanda Toland was supported by R01 CA215151.

CORSA: The CORSA study was funded by Austrian Research Funding Agency (FFG) BRIDGE (grant 829675, to Andrea Gsur), the "Herzfelder'sche Familienstiftung" (grant to Andrea Gsur) and was supported by COST Action BM1206. We kindly thank all individuals who agreed to participate in the CORSA study. Furthermore, we thank all cooperating physicians and students and the Biobank Graz of the Medical University of Graz.

CPS-II: The American Cancer Society funds the creation, maintenance, and updating of the Cancer Prevention Study-II (CPS-II) cohort. The authors thank the CPS-II participants and Study Management Group for their invaluable contributions to this research. The authors would also like to acknowledge the contribution to this study from central cancer registries

supported through the Centers for Disease Control and Prevention National Program of Cancer Registries, and cancer registries supported by the National Cancer Institute Surveillance Epidemiology and End Results program.

CRA: SNT was supported by National Institutes of Health grant R01 CA68535.

CRCGEN: Colorectal Cancer Genetics & Genomics, Spanish study was supported by Instituto de Salud Carlos III, co-funded by FEDER funds –a way to build Europe– (grants PI14-613 and PI09-1286), Agency for Management of University and Research Grants (AGAUR) of the Catalan Government (grant 2017SGR723), and Junta de Castilla y León (grant LE22A10-2). Sample collection of this work was supported by the Xarxa de Bancs de Tumors de Catalunya sponsored by Pla Director d'Oncología de Catalunya (XBTC), Plataforma Biobancos PT13/0010/0013 and ICOBIOBANC, sponsored by the Catalan Institute of Oncology.

DACHS: This work was supported by the German Research Council (BR 1704/6-1, BR 1704/6-3, BR 1704/6-4, CH 117/1-1, HO 5117/2-1, HE 5998/2-1, KL 2354/3-1, RO 2270/8-1 and BR 1704/17-1), the Interdisciplinary Research Program of the National Center for Tumor Diseases (NCT), Germany, and the German Federal Ministry of Education and Research (01KH0404, 01ER0814, 01ER0815, 01ER1505A and 01ER1505B). We thank all participants and cooperating clinicians, and everyone who provided excellent technical assistance.

HCCS: JCF was supported by the National Institutes of Health (grant numbers R01 CA155101, U01 HG004726, R01 CA140561, T32 ES013678, U19 CA148107, P30 CA014089)

Harvard cohorts (HPFS, NHS, PHS): HPFS is supported by the National Institutes of Health grants P01 CA055075, UM1 CA167552, U01 CA167552, R01 CA137178 (supporting ATC), R01 CA151993, R35 CA197735 and UM1 CA176726. NHS is supported by the National

Institutes of Health grants R01 CA137178, P01 CA087969, UM1 CA186107, R01 CA151993 (supporting SO), and R35 CA197735 (supporting SO). PHS is supported by the National Institutes of Health (R01 CA042182). We would like to thank the participants and staff of the HPFS, NHS and PHS for their valuable contributions as well as the following state cancer registries for their help: AL, AZ, AR, CA, CO, CT, DE, FL, GA, ID, IL, IN, IA, KY, LA, ME, MD, MA, MI, NE, NH, NJ, NY, NC, ND, OH, OK, OR, PA, RI, SC, TN, TX, VA, WA, WY. The authors assume full responsibility for analyses and interpretation of these data.

IWHS: This study was supported by NIH grants CA107333 (R01 grant awarded to P.J. Limburg) and HHSN261201000032C (N01 contract awarded to the University of Iowa).

MCCS cohort recruitment was funded by VicHealth and Cancer Council Victoria. The MCCS was further supported by Australian NHMRC grants 509348, 209057, 251553 and 504711 and by infrastructure provided by Cancer Council Victoria. Cases and their vital status were ascertained through the Victorian Cancer Registry (VCR) and the Australian Institute of Health and Welfare (AIHW), including the National Death Index and the Australian Cancer Database.

PLCO: Intramural Research Program of the Division of Cancer Epidemiology and Genetics and supported by contracts from the Division of Cancer Prevention, National Cancer Institute, NIH, DHHS. Funding was provided by National Institutes of Health (NIH), Genes, Environment and Health Initiative (GEI) Z01 CP 010200, NIH U01 HG004446, and NIH GEI U01 HG 004438. The authors thank the PLCO Cancer Screening Trial screening center investigators and the staff from Information Management Services Inc and Westat Inc. Most importantly, we thank the study participants for their contributions that made this study possible. Cancer incidence data have been provided by the District of Columbia Cancer Registry, Georgia Cancer Registry, Hawaii Cancer Registry, Minnesota Cancer Surveillance

System, Missouri Cancer Registry, Nevada Central Cancer Registry, Pennsylvania Cancer Registry, Texas Cancer Registry, Virginia Cancer Registry, and Wisconsin Cancer Reporting System. All are supported in part by funds from the Center for Disease Control and Prevention, National Program for Central Registries, local states or by the National Cancer Institute, Surveillance, Epidemiology, and End Results program. The results reported here and the conclusions derived are the sole responsibility of the authors.

SFCCR: The Seattle site of the Colon CFR Cohort (www.coloncfr.org), is supported in part by the National Cancer Institute (NCI) of the National Institutes of Health (NIH) Award U01 CA167551. Additional support for the SFCCR and the SFCCR Illumina HumanCytoSNP array were through NCI/NIH awards U01 CA074794 (to JDP) and U24 CA074794 and R01 CA076366 (to PAN). SG was supported by U01/U24 CA074783.

Support for case ascertainment was provided from the Surveillance, Epidemiology and End Results (SEER) Program of the NCI. The content of this manuscript does not necessarily reflect the views or policies of the NIH or SFCCR, nor does mention of trade names, commercial products, or organizations imply endorsement by the US Government, the SEER Program, or the CCFR. The authors would like to thank the study participants and staff of the Seattle Colon Cancer Family Registry and the Hormones and Colon Cancer study (CORE Studies).

WHI: The WHI program is funded by the National Heart, Lung, and Blood Institute, National Institutes of Health, U.S. Department of Health and Human Services through contracts HHSN268201100046C, HHSN268201100001C, HHSN268201100002C, HHSN268201100003C, HHSN268201100004C, and HHSN271201100004C. The authors thank the WHI investigators and staff for their dedication, and the study participants for making the program possible. A full listing of WHI investigators can be found at:

http://www.whi.org/researchers/Documents%20%20Write%20a%20Paper/WHI%20Investigator%20Short%20List.pdf
